# The Trihelix Genes in *Gardenia jasminoides* Evolution, Expression Profiles, and Potential Regulatory Functions in Growth, Development, and Multiple Stress Responses

**DOI:** 10.3390/biom16091298

**Published:** 2026-09-08

**Authors:** Jun Liu, Tingting Cheng, Jiefeng Kou, Lili Wang, Lixin Pei, Yan Yang, Conglong Lian, Jinxu Lan, Fei Zhang, Suiqing Chen

**Affiliations:** 1 School of Phamacy, Henan University of Chinese Medicine, Zhengzhou 450046, China; liujun_0325@hactcm.edu.cn (J.L.);; 2 Key Laboratory of Bamboo and Rattan Science and Technology, International Center for Bamboo and Rattan, National Forestry and Grassland Administration, Beijing 100102, China; 3 Changjiang Basin Ecology and Environment Monitoring and Scientific Research Center, Changjiang Basin Ecology and Environment Administration, Ministry of Ecology and Environment, Wuhan 430010, China; 4 State Key Laboratory of Utilization of Woody Oil Resource, Hunan Academy of Forestry, Changsha 414000, China; 5 China Collaborative Innovation Center of Research and Development on the Whole Industry Chain of Yu-Yao, Zhengzhou 450046, China

**Keywords:** *Gardenia jasminoides*, Trihelix gene family, genome-wide identification, expression characteristics, abiotic stress, qRT-PCR

## Abstract

Trihelix is a class of transcription factors unique to plants that play a major role in abiotic and biotic stress responses, seed isolate development, floral organ morphogenesis, and plant photomorphogenesis. Nevertheless, the Trihelix transcription factor family in *Gardenia jasminoides* (*G. jasminoides*) has not been systematically characterized. In this study, 11 *GjTrihelix* genes were identified from the *G. jasminoides* genome, unevenly distributed across five chromosomes, and can be classified into four subfamilies: GT-1, GT-2, SIP and SH4. Gene structure and functional motif analyses revealed high conservation within the same subfamily. Cis-acting element analysis showed that these genes are closely related to hormone responses, stress responses, and growth and development processes. Intraspecific synteny analysis showed a segmental duplication between *GjTrihelix-3* and *GjTrihelix-10*. Interspecific collinearity analysis revealed that *G. jasminoides* shared 21 collinear gene pairs with soybean, compared with five pairs with Arabidopsis and 13 with Populus, indicating greater syntenic block conservation between G. jasminoides and soybean. Transcriptome data analysis demonstrated distinct spatiotemporal expression specificity of this gene family. Several genes were constitutively expressed in fruits; *GjTrihelix-1* and *GjTrihelix-3* were predominantly expressed in green fruits, while *GjTrihelix-11* was highly expressed in red fruits. Under melatonin treatment, five *GjTrihelix* genes showed significant up-regulation and obvious transcriptional suppression of another five genes. Following infection by *Botryosphaeria dothidea*, *GjTrihelix-5* and *GjTrihelix-7* were progressively induced and peaked at 72 h. qRT-PCR results indicated that most *GjTrihelix* genes were highly expressed in leaves, while *GjTrihelix-11* was highly expressed in flowers. Most *GjTrihelix* genes were significantly down-regulated under NaCl, ABA, GA_3_ and IAA stresses. This study provides new insights into the potential association of the Trihelix transcription factor family in *G. jasminoides* growth, development, and stress adaptation, offering theoretical references for stress-resistant *G. jasminoides* breeding.

## 1. Introduction

The *Gardenia jasminoides* (*G. jasminoides*), also known as Gardenia, is an evergreen shrub belonging to the Gardenia genus in Rubiaceae with white jasmine-like flowers and a characteristic fragrance [1]. This species exhibits a broad geographic distribution and naturally occurs across temperate, subtropical, and tropical zones. In addition, *G. jasminoides* holds significant medicinal value, as its fruit, leaves, flowers, and roots can all be used in herbal medicine [2]. The dried mature fruit, recognized as a dual-purpose resource for both food and medicinal applications, was included in the first list of medicinal and edible substances promulgated by the Ministry of Health of China [3]. Accordingly, investigations into its growth, development and associated regulatory factors carry profound theoretical and practical significance. Transcription factors (TFs) play a key role in signal transduction, signal perception, and the transcriptional control of abiotic stress. Despite their essential biological functions, Trihelix gene family members in *G. jasminoides* remain largely uncharacterized to date.

Trihelix transcription factors (TFs) are plant-specific transcription factors ubiquitous across the plant kingdom, with high sequence conservation across diverse plant species. These proteins specifically bind to GT cis-elements within gene promoters, earning them the alternative name GT factors [4]. Each Trihelix TF harbors one or two conserved trihelix domains with a helix–loop–helix–loop–helix topology, a structural feature that mediates their specific recognition of light-responsive GT elements [5]. The first *Trihelix* family gene, *GT-1*, was originally isolated from pea (*Pisum sativum*) [6]. According to variations in conserved domain architecture and functional divergence, the Trihelix TF family is categorized into five subfamilies—GT-1, GT-2, SH4, SIP1 and GTγ—based on the amino acid composition of their functional domains. The functions of each subfamily are different. The GT-1 family controls gene expression [6]. The GT-2 family is involved in the response to light treatment [7,8]. The SH4 family plays a key role in cell differentiation [9]. The response to abiotic stressors, including salt stress and cold, dry conditions, is influenced by members of the GTγ subfamily [10]. Members of the SIP1 subfamily can significantly up-regulate expression levels in flowers and fruits [11]. By targeting diverse biological pathways, these subfamilies synergistically maintain the equilibrium between plant developmental programs and environmental adaptability.

Trihelix transcription factors (TTFs) are essential proteins that play a critical role in various aspects of plant growth and development. In general, trihelix family genes regulate floral organs [12], stomata [13], trichomes [14] (Yoo et al., 2010), embryos [15], and seed filling [16], and they are also involved in responses to cold stress [17], drought stress [18], and salt stress [19]. For instance, the transcript abundance of *GmGT-2*, a GT-2 subfamily transcription factor in soybean, is modulated through the phytochrome signaling pathway, and its transcriptional expression is markedly repressed by light signals [8]. In addition, the rice *OsGTγ-1* gene was strongly induced by high-salt stress, with its transcripts being activated within 1 h of salt treatment and reaching peak expression levels at 6 h; overexpression of this gene significantly enhances salt tolerance in rice [20]. In *Brassica napus*, overexpression of *BnSIP1-1* improved seed germination under osmotic pressure, salt, and ABA treatments; moreover, *BnSIP1-1* decreased the susceptibility of transgenic seedlings to osmotic pressure and ABA treatments [21]. In quinoa, *Cqtrihelix23* enhanced quinoa’s tolerance to salt stress by promoting root development, maintaining the antioxidant system, and reducing the Na+/K+ ratio [22]. In tomato, the GT-1 subfamily gene *ShCIGT* was induced by multiple abiotic stresses and ABA; overexpression of this gene improves plant tolerance to cold and drought while simultaneously lowering ABA sensitivity during post-germination growth stages [17]. In maize, *ZmTHX15*-overexpressing *Arabidopsis* exhibited stronger drought tolerance with less secondary oxidative damage and a higher photosynthetic rate [23]. In tomato, suppression of *SlGT31* resulted in delayed fruit ripening and decreased accumulation of total carotenoids and ethylene content [24]. Although trihelix TFs have been identified in some plant species, many questions remain that need further investigation regarding their specific functions and regulatory mechanisms, their interactions with other transcription factors, and the identification of downstream target genes.

To date, the Trihelix gene family has been identified and functionally characterized in numerous plant species; however, systematic investigations of this gene family in *G. jasminoides* are still lacking. In the present study, bioinformatics methods were adopted to identify Trihelix gene family members based on the genomic data of *G. jasminoides*. The protein characteristics, phylogenetic relationships, cis-acting elements in promoters, conserved motifs, and collinearity relationships were comprehensively analyzed to elucidate the fundamental characteristics of this gene family. Furthermore, transcriptome data and qRT-PCR were integrated to dissect the regulatory patterns of *Trihelix* genes in different organizations, biotic and abiotic stress. These findings offer insights into the adaptability of *G. jasminoides* to abiotic stress, potential quality enhancement, and the sustainable development of the *G. jasminoides* industry.

## 2. Materials and Methods

### 2.1. Identification of Trihelix Genes in G. jasminoides

The Trihelix protein sequences of *Arabidopsis* and rice were obtained from The *Arabidopsis* Information Resource (https://www.arabidopsis.org/) (accessed on 2 September 2026) and Rice Data (http://www.ricedata.cn/gene/) (accessed on 2 September 2026), respectively. The protein sequence was obtained from the *G. jasminoides* genome. The genome accession number was PRJNA477438 in the NCBI database (https://www.ncbi.nlm.nih.gov/) (accessed on 2 September 2026). To identify the non-redundant *Trihelix* genes in the *G. jasminoides* genome, we used the Hidden Markov Model (HMM) profile of the Myb/SANT-LIKE domain (PF13837) with the threshold e-value of 10^−5^. To confirm the Myb/SANT-LIKE domain in identified proteins, domain analysis was performed using the Interproscan tool (https://www.ebi.ac.uk/interpro/result/InterProScan/iprscan5-R20250208-075705-0178-93699187-p1m/internal-1739001403063-51-1/) (accessed on 2 September 2026) [25]. The resulting sequences were further subjected to NCBI Conserved Domain Search Service (CD Search) (https://www.ncbi.nlm.nih.gov/Structure/cdd/wrpsb.cgi) (accessed on 2 September 2026), Pfam database (http://pfam.sanger.ac.uk/) (2 September 2026) and SMART (http://smart.emblheidelberg.de/) [26]. Online tools were used to determine whether the candidate sequence contained the Trihelix. The pI/Mw tool from ExPASy (https://web.expasy.org/protparam/) (accessed on 2 September 2026) was used to estimate the physicochemical parameters, including the molecular weight (MW) and isoelectric point (pI) of each gene product, and the parameter (resolution) was set to average [27]. The 3D structures of the GjTrihelix proteins were modeled using SWISS-MODEL (https://swissmodel.expasy.org/) (accessed on 2 September 2026) [28].

### 2.2. Phylogenetic Analysis, Gene Structure, and Conserved Motif of Trihelix in G. jasminoides

A total of 11 Trihelix protein sequences from *G. jasminoides* were aligned using Clustal W with the default parameters. For the phylogenetic analysis of Trihelix protein from *G. jasminoides*, *Arabidopsis*, *Oryza sativa*, *Morus alba* and *Pinus massoniana* were used to construct the maximum-likelihood phylogenetic tree, and the Neighbor-Joining tree was retained for comparison. All sequence accession numbers are listed in Table S6.

To assess the exon-intron organization of the *Trihelix* genes, the entire *G. jasminoides* genome and coding sequences were obtained from the Genome Warehouse Database. The Gene Structure Display Server (GSDS) was used to illustrate the exon/intron structure for individual *GjTrihelix* genes by comparing their cDNAs and corresponding genomic DNA sequences. To identify the conserved motif structures shared by the GjTrihelix proteins, the online MEME (Multiple Expectation Maximization for Motif Elicitation) program (https://meme-suite.org/meme/tools/meme) (accessed on 2 September 2026) was used.

### 2.3. Cis-Regulatory Element Analysis in the Promoter Regions of Trihelix Genes in G. jasminoides

To identify the *cis*-elements in the promoter region of each *Trihelix* gene in *G. jasminoides*, the 2000 bp upstream sequences of the Trihelix-encoding DNA sequences were extracted from the available *G. jasminoides* genome data using the TBtools program, and possible cis-acting elements were analyzed using the PlantCARE database (http://bioinformatics.psb.ugent.be/webtools/plantcare/html/) (accessed on 2 September 2026). A total of 22 *cis*-elements were selected by removing *cis*-elements of functional ambiguity as well as core promoter elements. The *cis*-element distributions were visualized using TBtools-II v 2.467 [29].

### 2.4. Chromosome Location and Collinearity Analysis with Several Plant Species

The chromosome position of *Trihelix* genes was obtained from the *G. jasminoides* genome annotations using TBtools-II v 2.467. Dual Synteny Plotter software (https://github.com/CJ-Chen/ TBtools/) was used to analyze the homology of the *Trihelix* genes between *G. jasminoides* and *Arabidopsis*, *Populus trichocarpa*, rice, *Glycine max* and *Eucommia ulmoides*; TBtools software was used to visualize the obtained results, and the obtained Trihelix homologous pairs were highlighted.

### 2.5. Expression Patterns of GjTrihelixes in Different Tissues and Different Developmental Stages Based on RNA-Seq Data

Transcriptome data of *G. jasminoides* across different fruit developmental stages (sarcocarp of green fruit, peel of green fruit, sarcocarp of red fruit, and peel of red fruit) were obtained from the NCBI Sequence Read Archive (SRA) under the BioProject accession PRJNA688705. Transcriptome data for *Botryosphaeria dothidea* infection were obtained from BioProject PRJNA895943. Melatonin treatment with SRA accessions used PRJNA345428. The expression abundance of *GjTrihelix* genes was determined using their Fragments Per Kilobase of transcript per Million mapped reads (FPKM) values. Notably, some *GjTrihelix* genes exhibited an FPKM value of 0, which likely reflects expression levels below the detection limit of the current transcriptomic sequencing technology rather than absolute absence of expression. A heatmap of *GjTrihelix* expression was generated using TBtools, with expression values represented as log_2_(FPKM) to standardize the data. The FPKM values were shown in Supplementary Materials.

### 2.6. Plant Materials and Abiotic Stress Treatments

The *G. jasminoides* seedlings were cultured in a growth chamber with a 16 h light/8 h dark cycle at 22 °C/18 °C, 80% humidity, and watered with Hoagland nutrient solution every week. Different tissue expression patterns were analyzed using the roots, stems and leaves of 2-month-old natural seedlings of *G. jasminoides*. For salt-stress treatment, each pot containing *G. jasminoides* seedlings was irrigated with 100 mL of 200 mM NaCl solution into the vermiculite substrate. For hormone treatments, seedling leaves were evenly sprayed with hormone working solution (10 mL per plant) until complete leaf wetness was reached. Seedlings sprayed with solvent-containing distilled water served as the control group. All treatments were performed in the evening to avoid photodegradation of phytohormones. The leaves were sampled at 0, 24, and 48 h after hormone treatments, frozen in liquid nitrogen, and finally stored at −80 °C for RNA extraction. There were three independent replicates for each treatment.

### 2.7. RNA Extraction, cDNA Synthesis, and qRT-PCR Gene Expression Analysis

The RNA extraction kit (TIANGEN Biotechnologies, DP441, China) was used to extract total RNA according to the manufacturer’s instructions. A NanoDrop ND-2000 (Thermo Scientific, USA) spectrophotometer and 1% agarose gel electrophoresis were used to detect the RNA quality of all samples. For qRT-PCR, Premier 5.0 software was used to design the specific primers based on the coding sequence of *GjTrihelix* genes. The primers of all the *GjTrihelix* genes are listed in Supplementary Table S1. *18S* rRNA was used as the internal reference gene for quantitative analysis [30]. The qRT-PCR reactions were conducted on a Quant Studio 5 (ABI, USA) using the 2× Universal SYBR Green Fast qPCP Mix. The PCR reaction mixture (20 µL) contained 7.2 µL of cDNA, 10 µL of SYBR Advantage Premix, and 0.4 µL of each primer (10 nmol mL^−1^). The following amplification reactions were performed for qRT-PCR: 95 °C for 5 s, 60 °C for 30 s, 40 cycles. All reactions were performed in triplicate, both technically and biologically. The ∆CT and ∆∆CT values were calculated using the 2^−∆∆CT^ method. To ensure the accuracy of the data, we performed 3 biological replicates and 3 technical replicates.

### 2.8. Protein Interaction Network and GO Enrichment Analysis of GjTrihelix

The protein–protein interaction network of *G. jasminoides* was predicted using STRING (https://cn.string-db.org/) (accessed on 2 September 2026) based on *Arabidopsis* homologous proteins, with a confidence cutoff of 0.4. The resulting network was visualized in Cytoscape software 3.10.4 [31]. The same screening criteria were applied to identify motif-associated target genes, and gene regulatory networks were subsequently constructed in Cytoscape according to these screening outputs. For GO enrichment analysis, FDR correction was implemented for multiple-testing adjustment, and terms with adjusted *p* < 0.05 were defined as significantly enriched. TBtools-II v 2.467 was used to perform GO enrichment analysis on the upstream transcription factors and downstream target genes of *Trihelix* genes.

## 3. Results

### 3.1. Identification and Physicochemical Property Analysis of Trihelix Genes in G. jasminoides

In this study, a total of 11 Trihelix family genes were identified from the *G. jasminoides* genome and sequentially designated as *GjTrihelix-1* to *GjTrihelix-11*. The physicochemical properties of the encoded proteins were systematically analyzed using the ExPASy tool (https://web.expasy.org/protparam/) (accessed on 2 September 2026), and the detailed results are presented in Table 1. The analysis revealed that the amino acid length of GjTrihelix proteins ranged from 123 to 894 amino acids. Specifically, GjTrihelix-11 encoded the largest protein with 894 amino acids, while GjTrihelix-5 encoded the smallest one with only 123 amino acids. The molecular weight (MW) varied from 13,925.55 Da (GjTrihelix-5) to 98,917.37 Da (GjTrihelix-11). The isoelectric point (pI) ranged from 5.18 (GjTrihelix-7) to 9.14 (GjTrihelix-5). Among them, four proteins (GjTrihelix-2, GjTrihelix-5, GjTrihelix-9, and GjTrihelix-11) were alkaline proteins with pI values higher than 7.00, whereas the remaining seven proteins were acidic proteins with pI values below 7.00. The instability index analysis demonstrated that all GjTrihelix proteins had values greater than 40, ranging from 41.09 to 64.66, indicating that these proteins were unstable. The hydrophobicity and hydrophilicity analysis showed that the grand average of hydropathicity (GRAVY) values of all GjTrihelix proteins were negative, fluctuating between −1.178 (GjTrihelix-2) and −0.375 (GjTrihelix-11), which confirmed that all GjTrihelix proteins were hydrophilic. Subcellular localization prediction revealed that only GjTrihelix-11 was localized in chloroplasts, while the remaining ten GjTrihelix proteins were localized in the nucleus. In addition, GjTrihelix-4 was also found to be distributed in peroxisomes.

Secondary structure analysis of 11 GjTrihelix proteins was performed, and the results are shown in Table 2. All members of this family contained α-helix and random coil, while no β-turn was detected. The structural differences were mainly reflected in the existence of an extended strand: GjTrihelix-2, GjTrihelix-4, GjTrihelix-5, and GjTrihelix-7 lacked an extended strand, and the other seven members possessed this structural element. In terms of constituent proportion, the proportion of α-helix ranged from 25.44% to 54.51%, and that of random coil was between 45.49% and 68.37%. Further analysis of the proportion order of various secondary structures revealed that only GjTrihelix-2 had a higher proportion of α-helix than random coil, and the remaining ten members had random coil as the predominant structure, followed by α-helix. For the seven proteins containing extended strand, the proportion sequence was random coil > α-helix > extended strand. In conclusion, random coil was the main component of the secondary structure of GjTrihelix proteins. The differences in α-helix proportion and the presence or absence of extended strand among family members may provide a structural basis for the functional diversity of this protein family. Furthermore, to obtain more structural insights, the 3D structures of GjTrihelix proteins were also modeled. All these 3D structures had a similar structural composition to secondary structures (Figure S1).

### 3.2. Phylogenetic Analysis of Trihelix Gene Family in G. jasminoides

**Figure 1:**
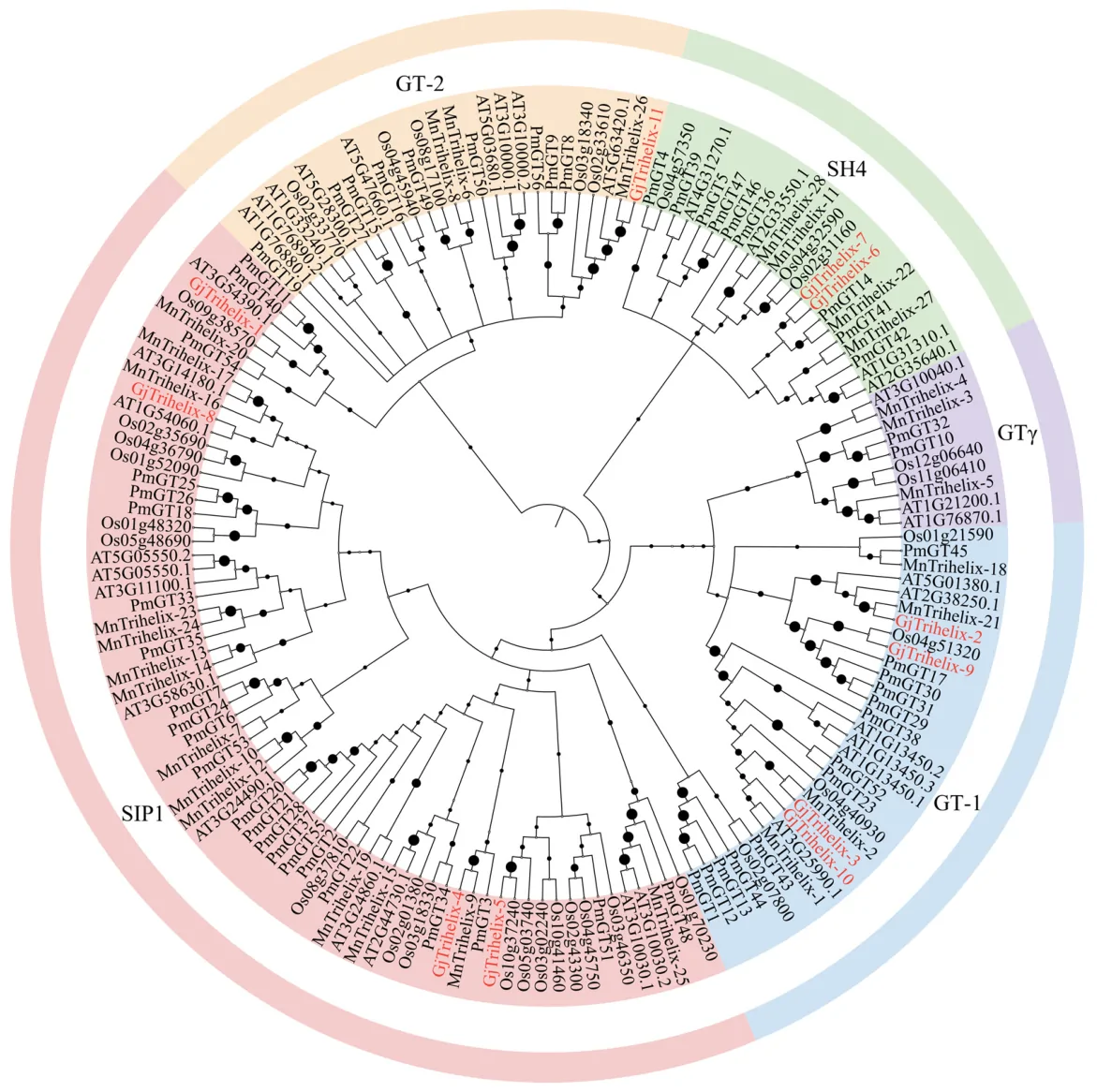
Phylogenetic tree of *Trihelix* proteins from *G. jasminoides*, *Arabidopsis thaliana*, *Oryza sativa*, *Morus alba* and *Pinus massoniana.* The red font represents the *G. jasminoides* Trihelix proteins. The size of black circles on tree nodes corresponds to bootstrap support values; larger circles indicate higher bootstrap values and greater confidence for the corresponding clade.

To examine the phylogenetic relationships among the *G. jasminoides Trihelix* genes, we constructed a phylogenetic tree with the Trihelix gene family members from *G. jasminoides* (11), *Arabidopsis* (34), rice (31), *Morus alba* (28) and *P. massoniana* (56). Based on phylogenetic analysis and previous Arabidopsis and rice studies, we classified 160 Trihelix sequences into five clades (Figure 1). Among these clades, the SIP1 subfamily contained the greatest number of homologs, followed by the GT-1, GT-2, SH4, and GTγ subfamilies. Phylogenetic clustering results revealed that the 11 identified *GjTrihelix* genes were unevenly distributed into four subfamilies, namely SIP1, GT-2, SH4 and GT-1. Notably, no GjTrihelix members clustered within the GTγ subfamily, implying that *G. jasminoides* lost GTγ-clade *Trihelix* genes during long-term evolutionary processes. The SIP1 and GT-1 subfamilies each possessed four GjTrihelix members, representing the two largest groups of gardenia *Trihelix* genes. The SH4 subfamily contained two paralogs, *GjTrihelix-6* and *GjTrihelix-7*, whereas the GT-2 subfamily contained only a single member, *GjTrihelix-11*. Within the SH4 clade, *GjTrihelix-6* and *GjTrihelix-7* clustered closely to form an independent monophyletic sub-branch. These results indicated that multiple species-specific gene duplication events occurred after the speciation of *G. jasminoides*. Notably, no GTγ-member was identified in the current *G. jasminoides* genome assembly, which may suggest a potential loss event, though we cannot fully rule out the possibility that it was caused by incomplete genome assembly or gene annotation.

### 3.3. Chromosome Localization and Collinearity Analysis of GjTrihelix Genes

**Figure 2:**
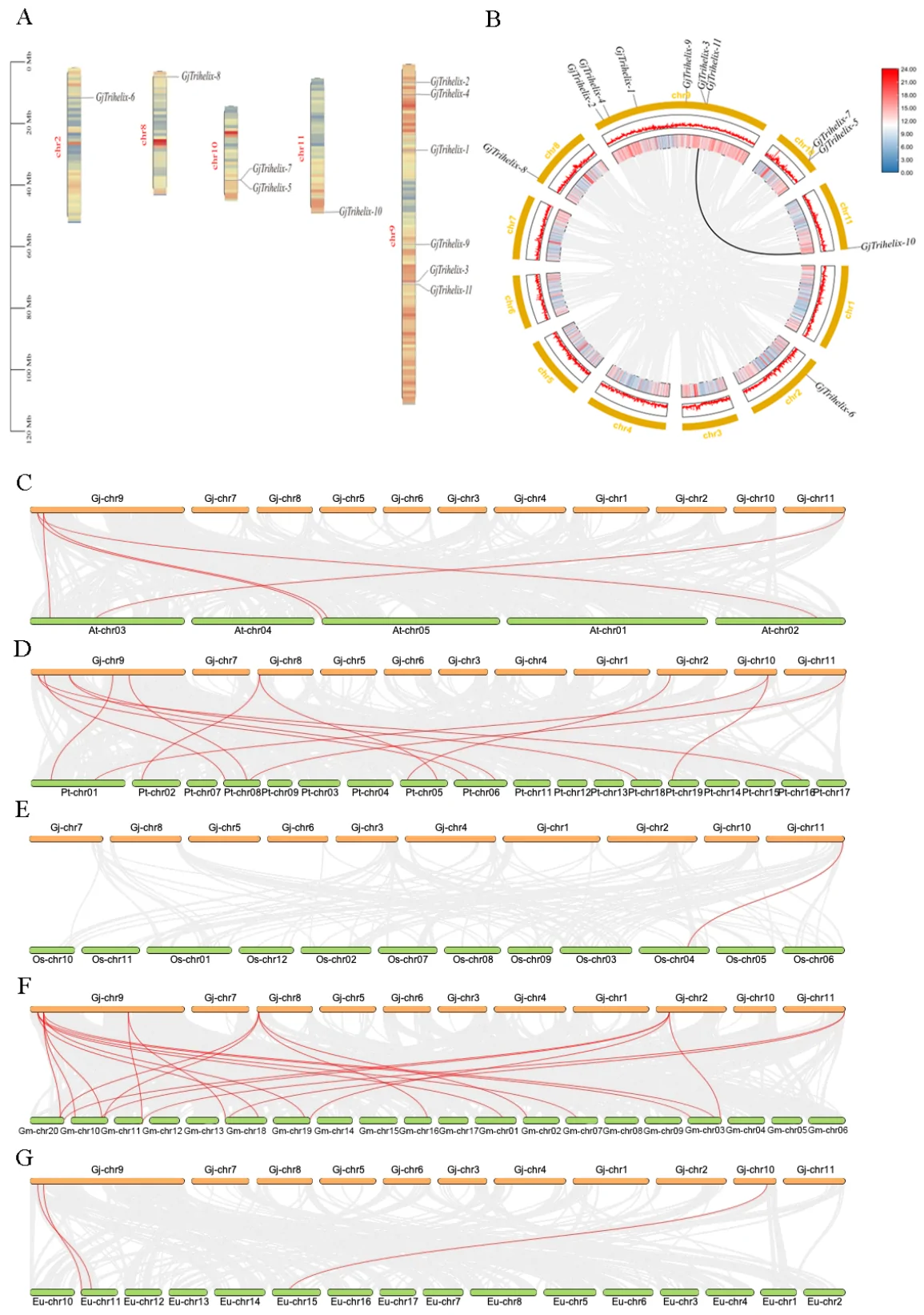
Chromosome localization and collinearity analysis of *GjTrihelix* genes. Note: (**A**) Chromosomal distribution of all identified *GjTrihelix* genes. (**B**) Intragenomic collinearity analysis of the *Trihelix* gene pairs in *G. jasminoides*. (**C**–**G**) Collinearity analysis of Trihelix gene families between *G. jasminoides* and *Arabidopsis* (**C**), poplar (**D**), rice (**E**), soybean (**F**), *Eucommia ulmoides* (**G**).

Chromosome localization analysis showed that 11 *GjTrihelix* genes were unevenly distributed on five chromosomes, including chr2, chr8, chr9, chr10 and chr11 (Figure 2A). Among them, chr9 contained the largest number of *GjTrihelix* genes with five members, followed by chr10 with two genes (*GjTrihelix-5* and *GjTrihelix-7*). In addition, only one *GjTrihelix* gene was distributed on chr2 (*GjTrihelix-6*), chr8 (*GjTrihelix-8*) and chr11 (*GjTrihelix-10*), respectively. To explore the evolutionary patterns of the *Trihelix* gene family in *G. jasminoides*, intra- and inter-specific collinearity analyses were performed in this study. The results of intraspecific collinearity analysis showed that among the 11 *GjTrihelix* genes, a segmental duplication event occurred between *GjTrihelix-3* and *GjTrihelix-10* (Figure 2B), suggesting that the Trihelix gene family has undergone a certain degree of expansion through duplication events in the *G. jasminoides* genome.

Interspecific collinearity analysis further revealed the homologous relationships between *GjTrihelix* genes and Trihelix family members from different plant species: five collinear gene pairs were identified between *G. jasminoides* and *Arabidopsis* (Figure 2C), 13 pairs with *Populus* (Figure 2D), one pair with rice (Figure 2E), three pairs with *Eucommia ulmoides* (Figure 2G), and the highest number of collinear gene pairs (21 pairs) with *Glycine max* (Figure 2F). *G. jasminoides* harbors more collinear *Trihelix* gene pairs with soybean than other investigated species, implying potential conservation of syntenic regions between the two species. In contrast, only one collinear gene pair was detected between *G. jasminoides* and rice, reflecting significant evolutionary divergence of the Trihelix family between monocots and dicots. Additionally, numerous collinear gene pairs were also identified between *G. jasminoides* and both *Populus* and *E. ulmoides*, suggesting strong homology and conservation of Trihelix family members among dicotyledonous plants.

### 3.4. Analysis of Cis-Acting Elements in the Promoter Region of the GjTrihelix Genes

**Figure 3:**
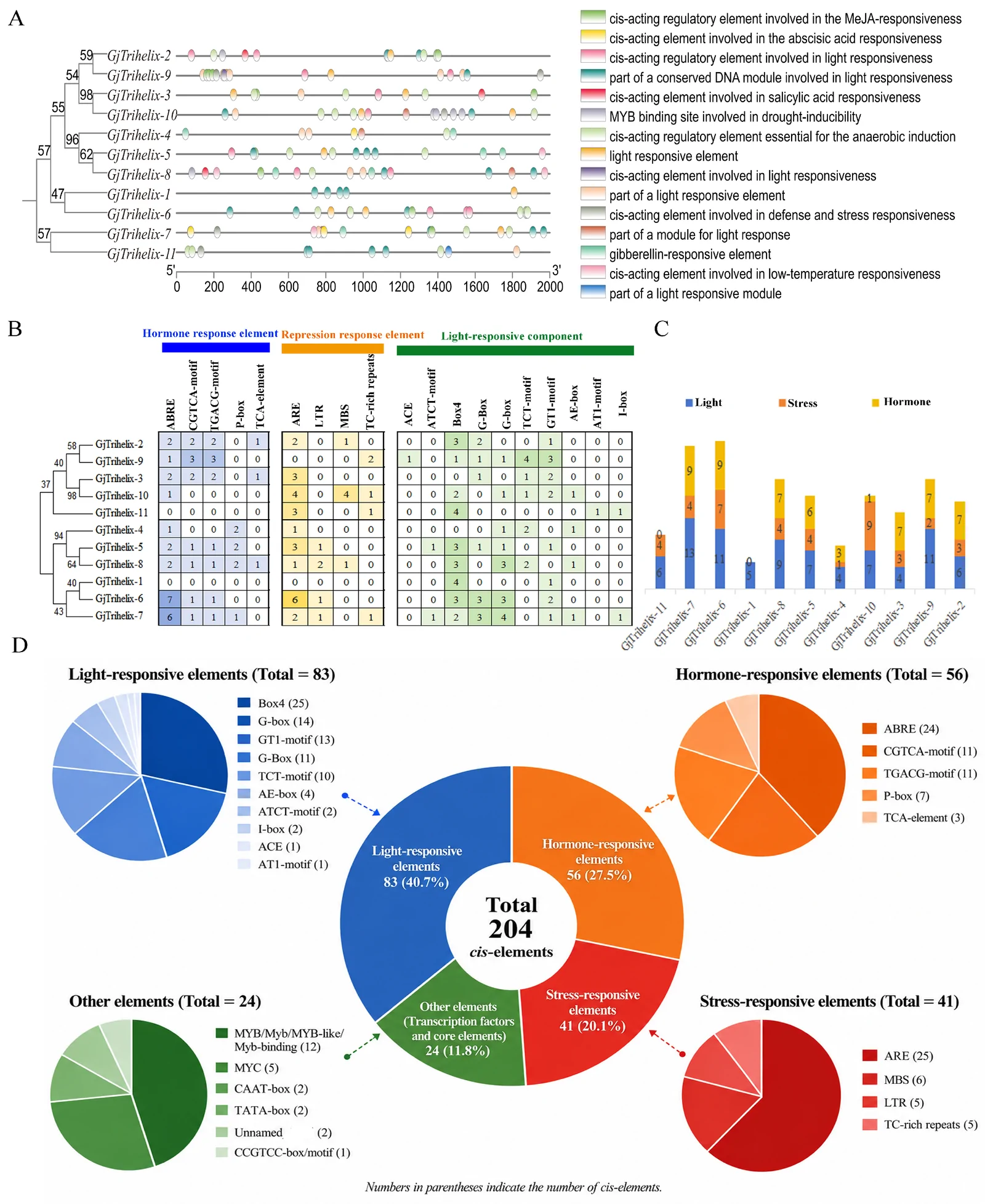
Analysis of promoter elements of the *G. jasminoides Trihelix* genes. Note: (**A**) Location map of promoter elements of *GjTrihelix* genes. (**B**) Number map of each element in *GjTrihelix* genes. (**C**) Total number map of each type of element in *GjTrihelix* genes. (**D**) Proportion map of each element in four categories of elements.

The cis-acting elements of the promoter sequences within 2000 bp upstream of the initiation codon of 11 *GjTrihelix* genes were analyzed (Figure 3). A total of 180 *cis*-acting elements were identified, which could be classified into three categories based on functional characteristics: hormone-responsive elements (83), stress-responsive elements (41), and light-responsive elements (56). Among the hormone-responsive elements, ABRE was the most abundant element with 24 copies, accounting for 42.86% of this category. CGTCA-motif and TGACG-motif had the same quantity (11), each accounting for 19.64% of hormone-responsive elements. Stress-responsive elements contained ARE, LTR, MBS, TC-rich repeats and other functional motifs. ARE was the predominant stress-responsive element with 25 copies, occupying 60.98% of this category. A total of ten types of light-responsive elements were detected, such as ACE, ATCT-motif, Box4, G-Box and TCT-motif. Box4 was the most abundant light-responsive element (25 copies), accounting for 30.12% of this category. Notably, the promoter of *GjTrihelix-6* was enriched with ABRE and ARE elements, while *GjTrihelix-7* contained a high number of ABRE elements. These are putative cis-elements predicted by bioinformatics of *GjTrihelix* genes, and their hormone- or stress-responsive activity needs further experimental validation.

### 3.5. Analysis of the Conserved Motifs, Domains and Gene Structure of the GjTrihelix Genes

**Figure 4:**
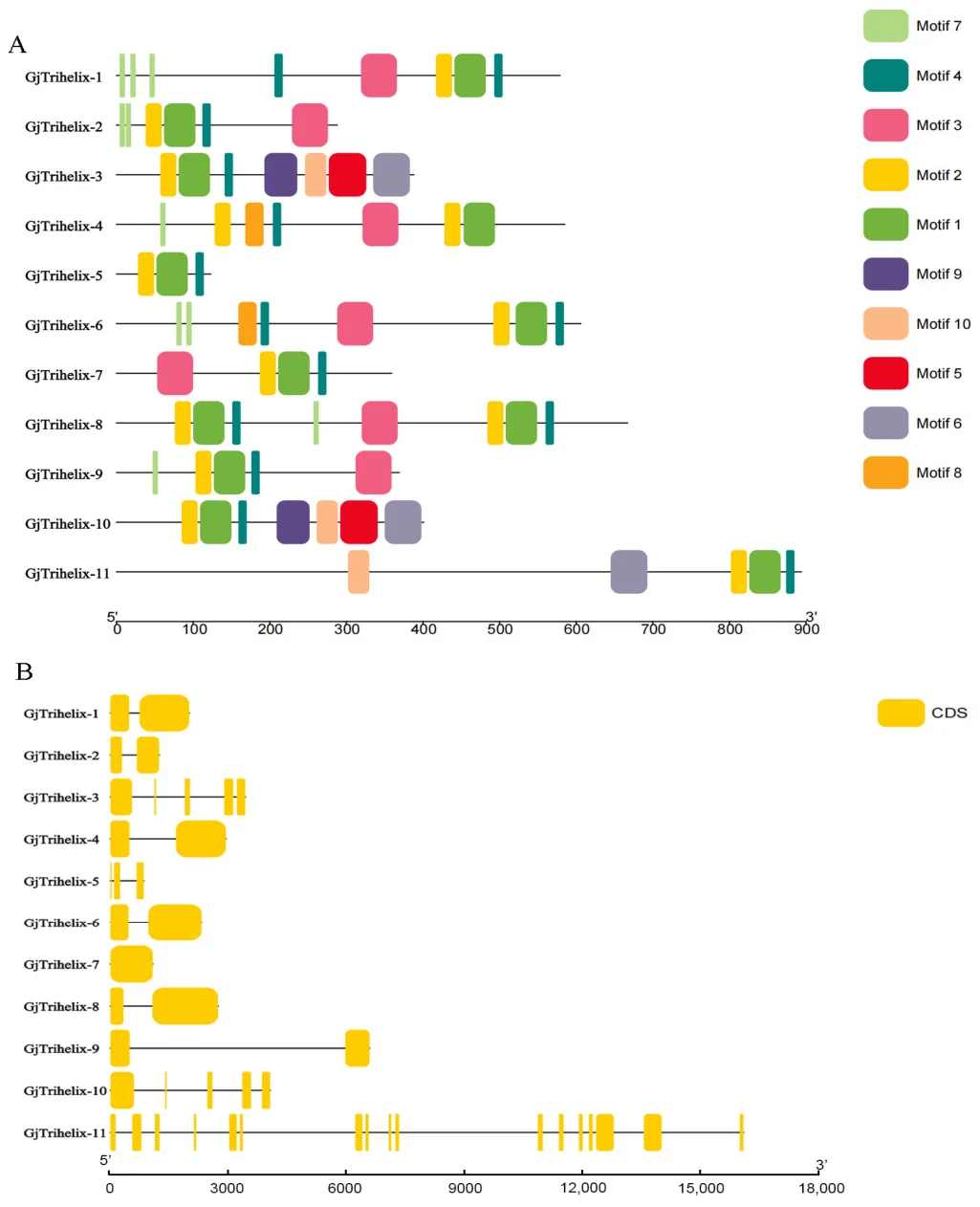
Analysis of conserved motifs, domain and gene structures of *GjTrihelix* genes. Note: (**A**) Conserved motifs of *GjTrihelix*. (**B**) Gene structure of *GjTrihelix*.

To clarify the sequence conservation characteristics of the *Trihelix* gene family in *G. jasminoides*, systematic analyses of conserved motifs, conserved domains and gene structures were performed on family members. A total of ten conserved motifs were predicted and designated as Motif 1 to Motif 10. The results of conserved motif analysis (Figure 4A) revealed that all 11 *GjTrihelix* genes contained Motif 1, Motif 2 and Motif 4, indicating that the three motifs were core-conserved elements of this gene family and likely play crucial roles in maintaining the basic functions of *Trihelix* genes. Furthermore, Motif 5, Motif 6, Motif 9 and Motif 10 were uniquely distributed in the GT-1 subfamily, and the motif composition varied distinctly among different subfamilies. It was inferred that the differential distribution of conserved motifs provided a crucial molecular basis for the functional differentiation of *GjTrihelix* genes. Gene structure analysis (Figure 4B) showed obvious differences in exon numbers among *GjTrihelix* genes. *GjTrihelix-11* had the largest number of exons (17), *GjTrihelix-3* and *GjTrihelix-10* each contained five exons, *GjTrihelix-5* contained three exons, *GjTrihelix-7* had only one exon, and the remaining members possessed two exons.

### 3.6. Expression Patterns of GjTrihelix During the Developmental Stages of G. jasminoides Fruits

**Figure 5:**
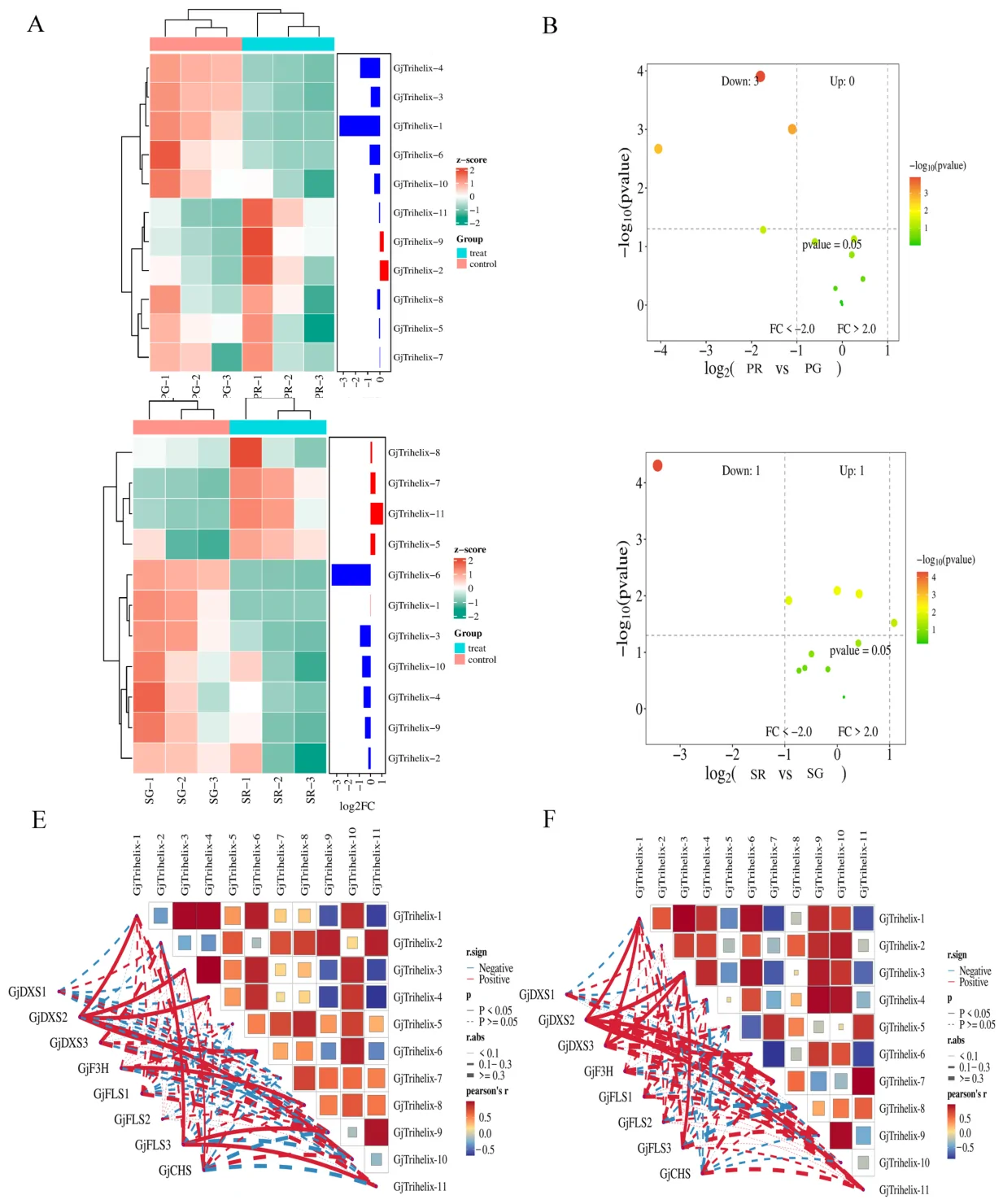
Analysis of the expression patterns of *Trihelix* during different developmental stages of *G. jasminoides* fruit. Note: (**A**) Expression clustering heatmap of Trihelix in peel. (**B**) Volcano plot showing differential gene expression between PR and PG. (**C**) Expression clustering heatmap of Trihelix in sarcocarp. (**D**) Volcano plot showing differential gene expression between SR and SG. (**E**) Correlation analyses between *GjTrihelix* genes and flavonoid biosynthesis genes in peel. (**F**) Correlation analyses between *GjTrihelix* genes and flavonoid biosynthesis genes in sarcocarp. PG: Peel of green fruit. PR: Peel of red fruit. SG: Sarcocarp of green fruit. SR: Sarcocarp of red fruit.

To explore the potential correlation between the *GjTrihelix* gene family and fruit development in *G. jasminoides*, the expression patterns of *GjTrihelix* genes were systematically analyzed based on transcriptome data of peel and pulp from green and red fruits. The results show that the expression levels of *GjTrihelix* members varied significantly among different fruit colors and tissues (Figure 5A,C). All genes were classified into four categories according to their expression characteristics. Constitutive highly expressed genes, including *GjTrihelix-5*, *GjTrihelix-7* and *GjTrihelix-8*, maintained high expression levels in both peel and pulp of green and red fruits. Constitutive lowly expressed genes (*GjTrihelix-2*, *GjTrihelix-4* and *GjTrihelix-9*) exhibited low expression abundance in all tested samples. Four genes (*GjTrihelix-1*, *GjTrihelix-3*, *GjTrihelix-6* and *GjTrihelix-10*) were predominantly expressed in green fruits, with significantly higher expression in green fruit peel and pulp than in red fruits; these four genes may be mainly involved in the early development of *G. jasminoides* fruits. Notably, *GjTrihelix-11* was significantly up-regulated in the peel and pulp of red fruits, suggesting that *GjTrihelix-11* is a candidate regulator potentially associated with pigment accumulation during fruit ripening. However, its specific function needs to be further validated. Volcano plots were used to identify differentially expressed GjTrihelix genes. In the PR-vs-PG comparison, zero genes were significantly up-regulated, whereas three genes were significantly down-regulated. For SR-vs-SG, one gene was significantly up-regulated and one gene was significantly down-regulated (Figure 5B,D).

To elucidate the potential regulatory relationship between Trihelix transcription factor and genes involved in the flavonoid biosynthesis pathway, we constructed a gene co-expression network comprising flavonoid biosynthesis structural genes (including *GjDXSs*, *GjF3H*, *GjFLSs*, and *GjCHSs*) and 11 *GjTrihelix* family genes based on transcriptomic data from *G. jasminoides* peel and sarcocarp tissues (Figure 5E,F). Co-expression relationships were screened with a threshold of absolute correlation coefficient |r| ≥ 0.60 and a significance level of *p* < 0.05. Network analysis revealed significant correlations between *GjTrihelix* genes and the core flavonoid biosynthesis structural genes. In the peel tissue, nine co-expression pairs were detected between the eight flavonoid structural genes and 11 *GjTrihelix* genes. Among these, *GjTrihelix-2* and *GjTrihelix-6* showed the highest correlation degrees, each associating with two flavonoid biosynthesis genes. Meanwhile, five *GjTrihelix* genes (*GjTrihelix-1*, *GjTrihelix-3*, *GjTrihelix-4*, *GjTrihelix-10*, and *GjTrihelix-11*) were individually correlated with one flavonoid structural gene. In the sarcocarp-derived co-expression network, a total of eight co-expression pairs were identified between eight flavonoid structural genes and 11 *GjTrihelix* genes. Statistical analysis demonstrated that *GjTrihelix-1* exhibited the strongest correlation, interacting with two flavonoid biosynthesis genes. Additionally, four *GjTrihelix* members (*GjTrihelix-3*, *GjTrihelix-6*, *GjTrihelix-7*, and *GjTrihelix-11*) were each co-expressed with a single flavonoid structural gene. Collectively, these results indicate that *GjTrihelix* family genes are potentially involved in the flavonoid biosynthesis pathway in *G. jasminoides.* It should be noted that the co-expression network was inferred from a limited number of green- and red-fruit samples. Correlation-based associations are exploratory and do not prove causal regulatory relationships. Further experiments are required to verify the potential functions of *GjTrihelix* genes in flavonoid biosynthesis.

### 3.7. Analysis of the Expression Pattern of GjTrihelix Under Melatonin Treatment

**Figure 6:**
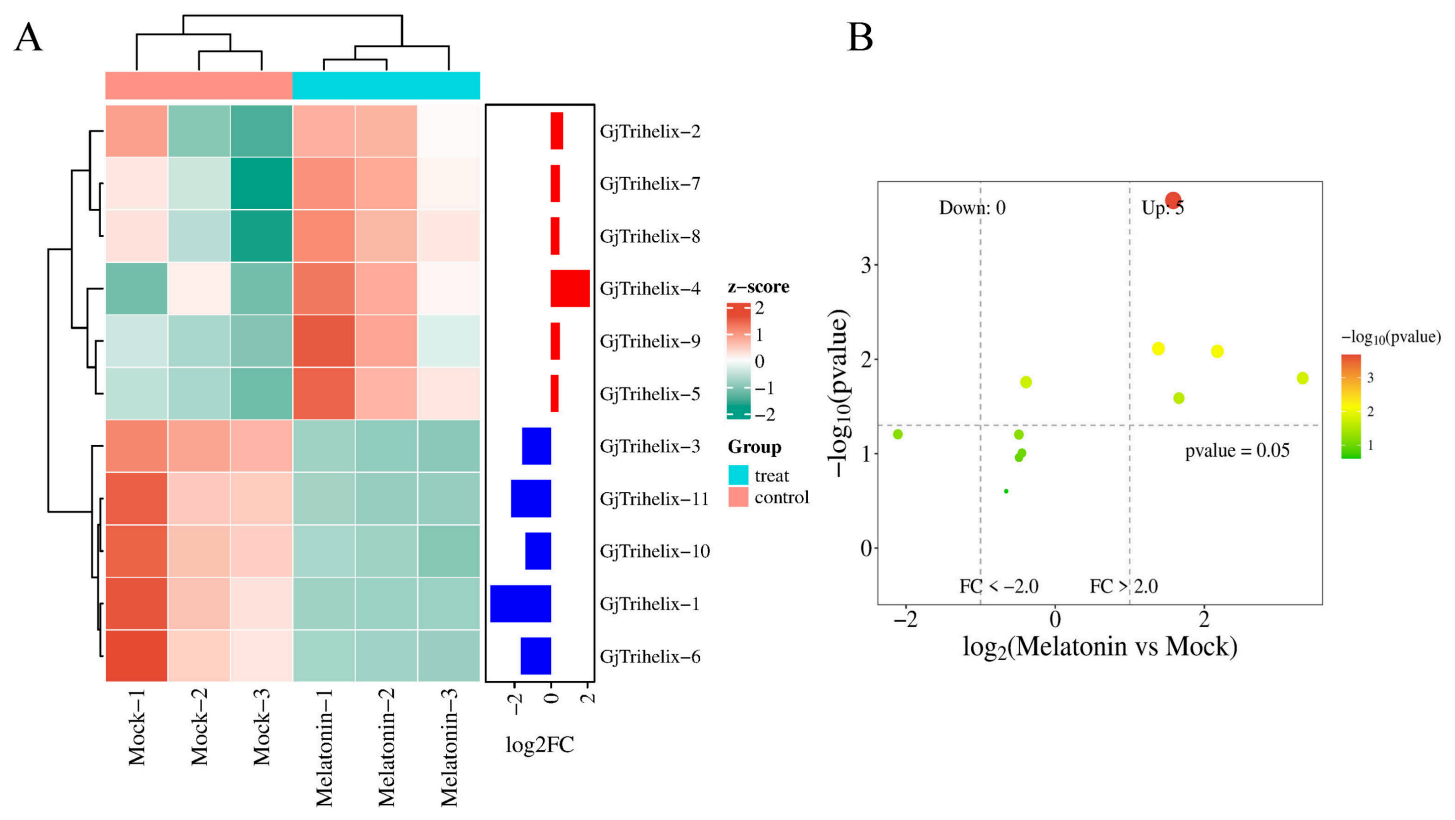
The expression pattern of *GjTrihelix* in response to melatonin treatment. Note: (**A**) Expression clustering heatmap normalized. Red/green colors denote high/low expression, and the side bar visualizes log_2_ fold changes (red: up-regulation; blue: down-regulation). (**B**) Volcano plot showing differential gene expression between melatonin-treated and Mock samples. The x-axis indicates log_2_(fold change), and the y-axis shows −log_10_(*p*-value). Vertical dashed lines mark the 2-fold expression threshold, while the horizontal dashed line represents the significance cutoff at *p* = 0.05. Five genes were significantly up-regulated, with no significantly down-regulated genes detected; dot colors reflect the magnitude of statistical significance.

Melatonin (MT) serves as a signaling molecule to regulate growth and development while enhancing plant stress resistance. Recent studies have demonstrated that, as both a growth regulator and antioxidant, melatonin can mitigate the physiological damage caused by abiotic stress by interacting with other plant hormones. It protects plants against oxidative stress, delays leaf senescence, and enhances the activities of antioxidant enzymes, thereby activating the antioxidant defense system [32]. To dissect the regulatory mechanism of exogenous melatonin on the expression of *GjTrihelix* family genes in *G. jasminoides*, leaf samples with melatonin treatment and untreated control were collected for transcriptome sequencing, and the expression characteristics of 11 *GjTrihelix* family members were systematically analyzed. The results revealed significant differential responses of *GjTrihelix* family genes to exogenous melatonin induction (Figure 6). Among them, the transcript levels of *GjTrihelix-2*, *GjTrihelix-5*, *GjTrihelix-7*, *GjTrihelix-8* and *GjTrihelix-9* were markedly up-regulated after melatonin treatment. As highly expressed genes, *GjTrihelix-5* and *GjTrihelix-7* exhibited a high accumulation of transcripts in melatonin-treated samples. In contrast, *GjTrihelix-1*, *GjTrihelix-3*, *GjTrihelix-6*, *GjTrihelix-10* and *GjTrihelix-11* showed distinct negative responses to melatonin. Compared with the blank control group, melatonin treatment suppressed the transcription of these genes, with *GjTrihelix-1* and *GjTrihelix-11* displaying the most prominent inhibitory effects. In addition, *GjTrihelix-4* maintained an extremely low basal expression level in all experimental samples, with no significant expression differences between the treatment and control groups. This result implies that *GjTrihelix-4* may be transcriptionally insensitive to exogenous melatonin.

### 3.8. Expression Patterns of Trihelix Genes in Response to Botryosphaeria dothidea Infection in G. jasminoides Leaves

**Figure 7:**
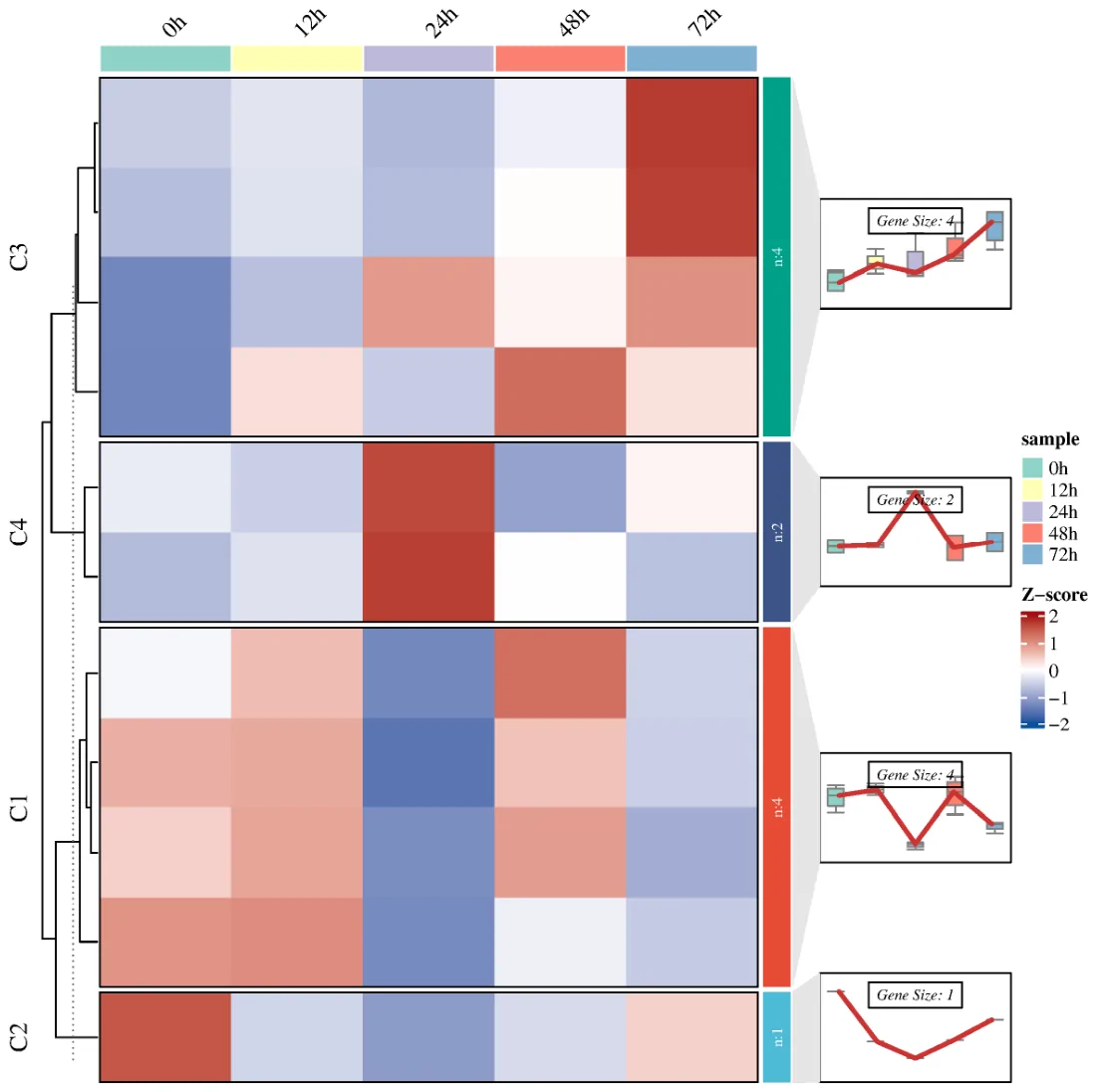
Expression profiles of *GjTrihelix* genes in *G. jasminoides* leaves in response to *B. dothidea* infection. Note: Rows represent individual *GjTrihelix* genes, columns represent infection time points, and color gradients indicate normalized Z-score values of transcript abundance (red = high expression, blue = low expression). Genes were divided into four subclusters (C1–C4) according to their temporal expression patterns. Small line charts on the right illustrate the average expression trend of genes within each subcluster, with n indicating the number of genes contained in each cluster.

To clarify the potential regulatory roles of *Trihelix* family genes in the defense response of *G. jasminoides* against fungal disease, we systematically profiled their temporal expression dynamics in leaf tissues at four critical time points (0, 12, 24, 48, and 72 h) following inoculation with *Botryosphaeria dothidea*. Based on normalized Z-score expression patterns, all genes were classified into four distinct subclusters (C1–C4) with unique time-dependent expression trajectories (Figure 7), demonstrating obvious functional differentiation of *GjTrihelix* paralogs during fungal infection. Subcluster C1 contained four genes, namely *GjTrihelix-2*, *GjTrihelix-3*, *GjTrihelix-6* and *GjTrihelix-11*. Genes in this cluster were moderately induced at early infection stages, were sharply suppressed at 24 h, and reached secondary expression peaks at mid-late infection timepoints. Specifically, *GjTrihelix-3* achieved its maximum FPKM of 21.98 at 12 h and dropped to the lowest value (FPKM = 12.14) at 24 h; *GjTrihelix-2* peaked at 12 h (FPKM = 2.22) with a minimum transcript level of 0.69 at 24 h; *GjTrihelix-6* exhibited the highest expression at 48 h (FPKM = 14.18); *GjTrihelix-11* displayed the peak FPKM of 55.43 at 48 h after a striking decline at 24 h (FPKM = 13.84). The average expression trend of C1 genes showed an early mild up-regulation, a universal transcriptional depression at 24 h, and re-induction at 48 h. Subcluster C2 harbored a single gene, *GjTrihelix-1*, which possessed the highest basal expression at 0 h (FPKM = 3.07) and was continuously suppressed after pathogen inoculation, with the minimal transcript accumulation detected at 24 h (FPKM = 0.90). This expression signature indicated that *GjTrihelix-1* was negatively regulated by *B. dothidea* infection. Subcluster C4 included two genes (*GjTrihelix-4* and *GjTrihelix-10*) that were specifically and strongly activated at 24 h. *GjTrihelix-10* reached its global maximum FPKM of 27.04 at 24 h, while its transcript level decreased to the minimum value of 15.59 at 48 h. *GjTrihelix-4* maintained extremely low expression across all time points and only showed a slight expression burst at 24 h (FPKM = 0.29). Subcluster C3 comprised four genes (*GjTrihelix-5*, *GjTrihelix-7*, *GjTrihelix-8*, and *GjTrihelix-9*) characterized by sustained up-regulation throughout infection and prominent late-stage expression peaks at 48 or 72 h. Among them, *GjTrihelix-7* exhibited the highest overall transcription abundance across the entire family, with FPKM values climbing continuously from 0 h to a record peak of 162.99 at 72 h, even though its expression was transiently reduced at 24 h (FPKM = 56.04). *GjTrihelix-5* also showed a persistent rising expression trend and peaked at 72 h (FPKM = 126.43). *GjTrihelix-8* reached its expression maximum at 48 h (FPKM = 47.63), whereas *GjTrihelix-9* attained the highest transcript level at 72 h (FPKM = 7.38). The average trend of C3 genes presented progressive induction and dominant high expression at the late infection stage (72 h). Statistical summary of peak expression timepoints revealed a clear sequential activation pattern across the infection gradient: two *GjTrihelix* genes peaked at 12 h, two genes peaked at 24 h, three genes reached maximum transcript abundance at 48 h, and three genes showed their highest expression at 72 h. Notably, seven out of 11 *GjTrihelix* members displayed their lowest FPKM values at 24 h, which implies the occurrence of widespread transcriptional repression at this key intermediate time point during *B. dothidea* colonization. Collectively, these stage-biased and divergent expression patterns indicate transcriptional differentiation among GjTrihelix family members. These dynamically expressed genes serve as reliable candidate resources for further dissecting the molecular defense mechanisms of *G. jasminoides* against *B. dothidea*.

### 3.9. Expression Analysis of GjTrihelix Genes in Various Tissues by qRT-PCR

**Figure 8:**
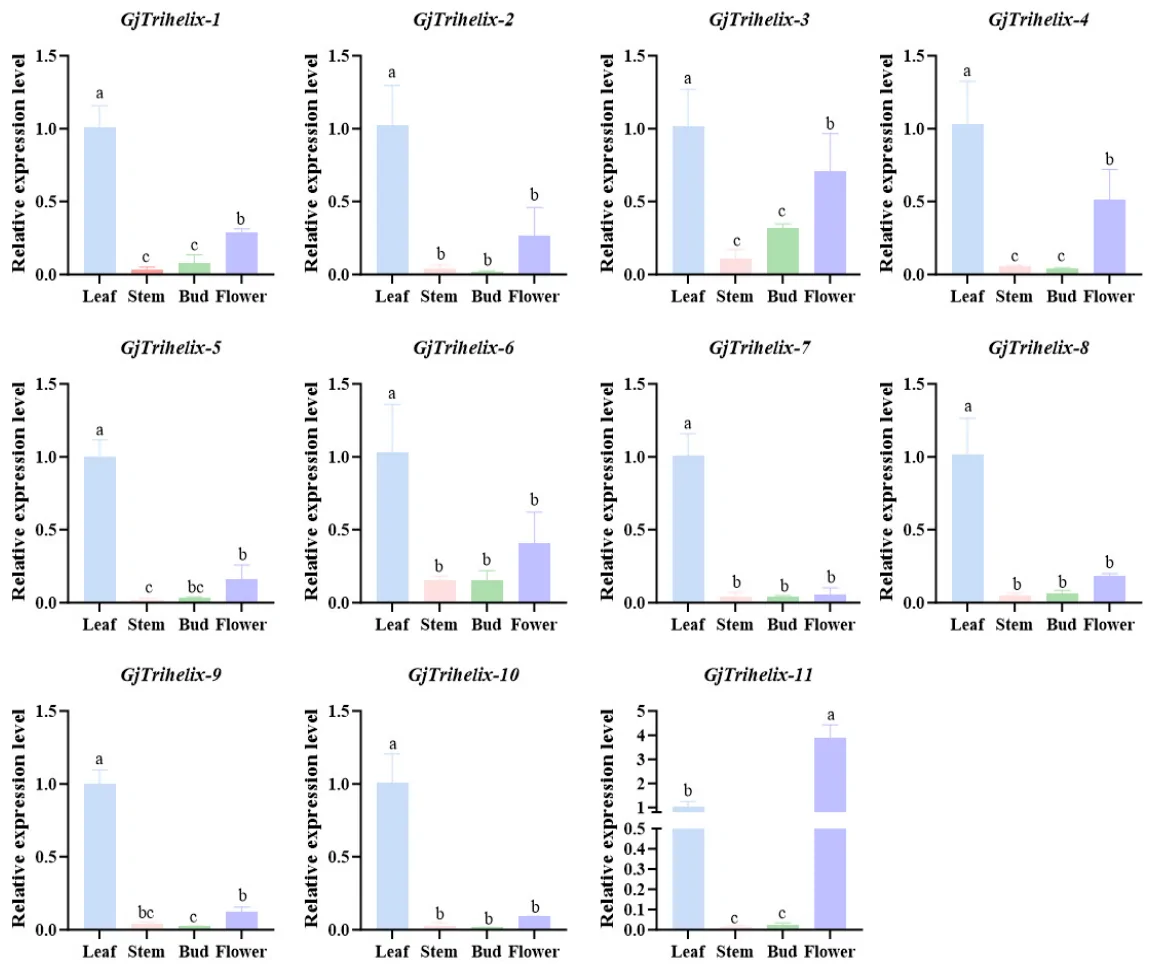
Expression patterns of *GjTrihelix* genes in leaves, stems, buds, and flowers of *G. jasminoides*. Note: Different lowercase letters (a, b, c) above the bars indicate statistically significant differences (*p* < 0.05) according to Duncan’s multiple range test. Bars sharing the same letter are not significantly different. Results are expressed as mean ± standard deviation (SD) (*n* = 3). Error bars represent the SD of three independent replicates.

To characterize the tissue-specific expression patterns of *GjTrihelix* genes, we quantified their transcript levels in four tissues, including leaf, stem, bud and flower, using qRT-PCR. The results showed that the expression patterns of different genes vary significantly in different tissues (Figure 8). Among the 11 *GjTrihelix* genes, 10 of them had the highest expression levels in the leaves, accounting for 90.9% of the total number of genes detected, suggesting that this family may play a central role in leaf development or function maintenance. Notably, only *GjTrihelix-11* exhibited predominant expression in flowers, with a relative expression level of approximately 3.87, which was markedly higher than that in leaves (1.28), stems (0.02), and buds (0.01), indicating a potential specialized function in floral organ development. No genes displayed their highest expression levels in stems or buds, implying that these tissues are not the primary sites of action for this gene family. Regarding the tissues with the lowest transcript abundance, four genes (*GjTrihelix-3*, *GjTrihelix-5*, *GjTrihelix-6*, and *GjTrihelix-8*) exhibited their lowest expression levels in stems, accounting for 36.4% of the total members. Among them, *GjTrihelix-5* showed the lowest expression, with transcript levels approximately 0.017-fold of that in leaves, followed by *GjTrihelix-8* (about 0.050-fold of leaf expression). The remaining seven *GjTrihelix* genes (63.6%) displayed their lowest expression levels in buds, among which *GjTrihelix-2* had the lowest transcript abundance (approximately 0.008-fold of leaf expression), followed by *GjTrihelix-11* (approximately 0.011-fold). Collectively, these findings demonstrate that members of the *GjTrihelix* family exhibit pronounced tissue-specific expression patterns, implying potential functional divergence of these genes across various tissues in *G. jasminoides*, which requires further experimental validation.

### 3.10. Expression Analysis of GjTrihelix Under Salt Stress

**Figure 9:**
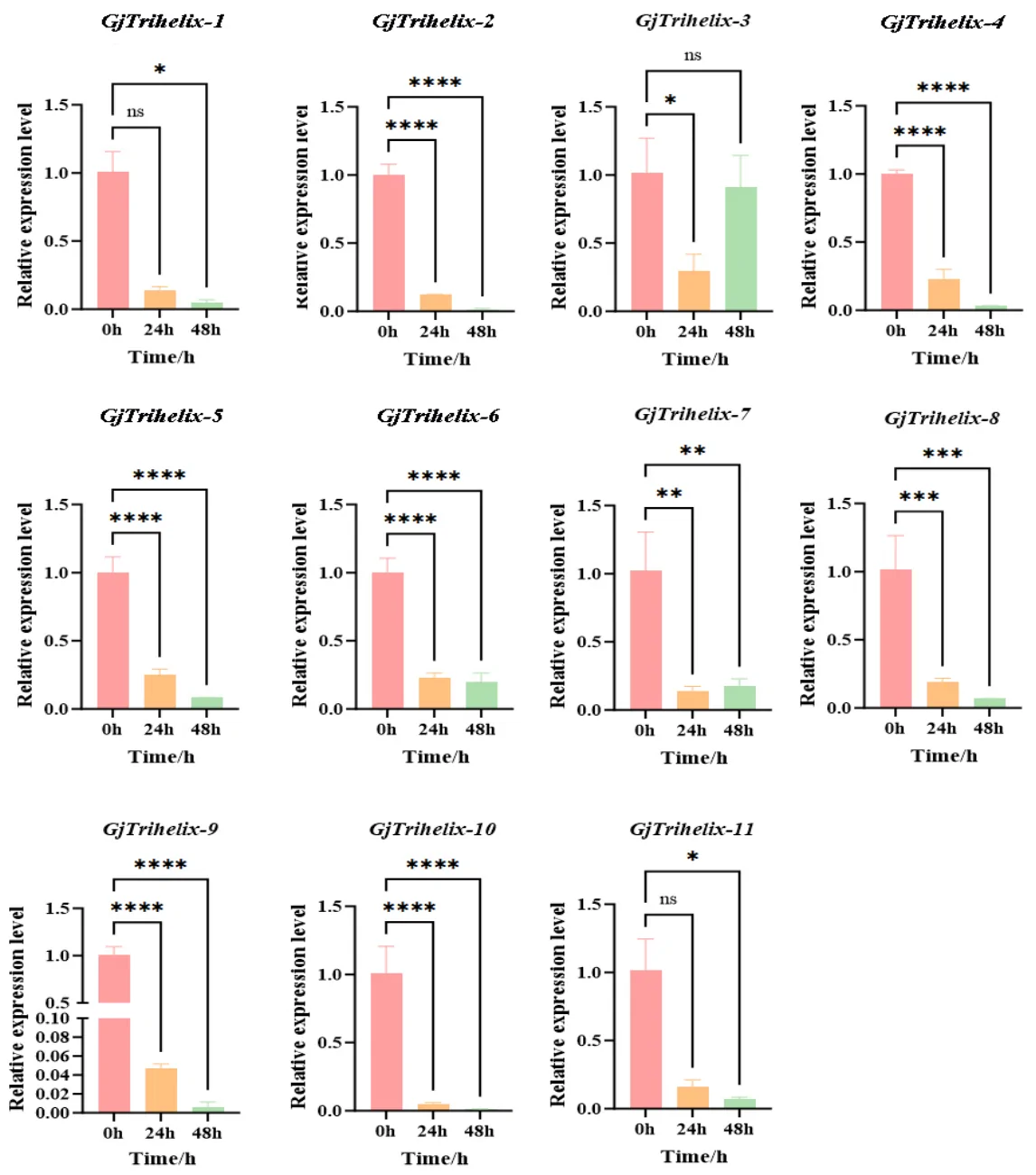
The expression patterns of *GjTrihelix* genes under NaCl treatments. qRT-PCR data were normalized using *18S* as the reference gene. Results are expressed as mean ± SD (*n* = 3). The * indicates significant differences compared to 0 h, as determined by *t*-test (* *p* < 0.05, ** *p* < 0.01, *** *p* < 0.001, **** *p* < 0.0001), ns represents no significant difference. Error bars represent the SD of three independent replicates.

To elucidate the response patterns of the *GjTrihelix* gene family to salt stress, the expression levels of 11 family members were examined by qRT-PCR at 0, 24, and 48 h following NaCl treatment (Figure 9). The results showed that, with the exception of *GjTrihelix-3*, the remaining ten genes exhibited a continuous decline in expression over the course of stress treatment. Among them, *GjTrihelix-9* and *GjTrihelix-10* were the most severely repressed, with transcript levels decreasing to 0.007-fold and 0.0008-fold of the control at 48 h, respectively, with nearly complete transcriptional repression. Notably, *GjTrihelix-3* displayed a distinct expression pattern: its expression was significantly down-regulated to 0.44-fold of the control at 24 h but rebounded markedly at 48 h, approaching the control level, implying a potential role in the adaptive regulation during the late phase of salt stress. Additionally, *GjTrihelix-7* also showed a slight recovery at 48 h (approximately 0.15-fold of the control), while the remaining members maintained relatively low expression levels throughout the treatment. Collectively, these findings indicate that the majority of *GjTrihelix* genes are subjected to strong transcriptional repression under salt stress, with only *GjTrihelix-3* exhibiting a phase-specific recovery in transcript level. These expression patterns imply that the *GjTrihelix* family may be involved in the salt-stress response of *G. jasminoides*, and their exact regulatory roles remain to be experimentally validated.

### 3.11. Transcriptional Responses of GjTrihelix Genes to Hormonal Treatments

**Figure 10:**
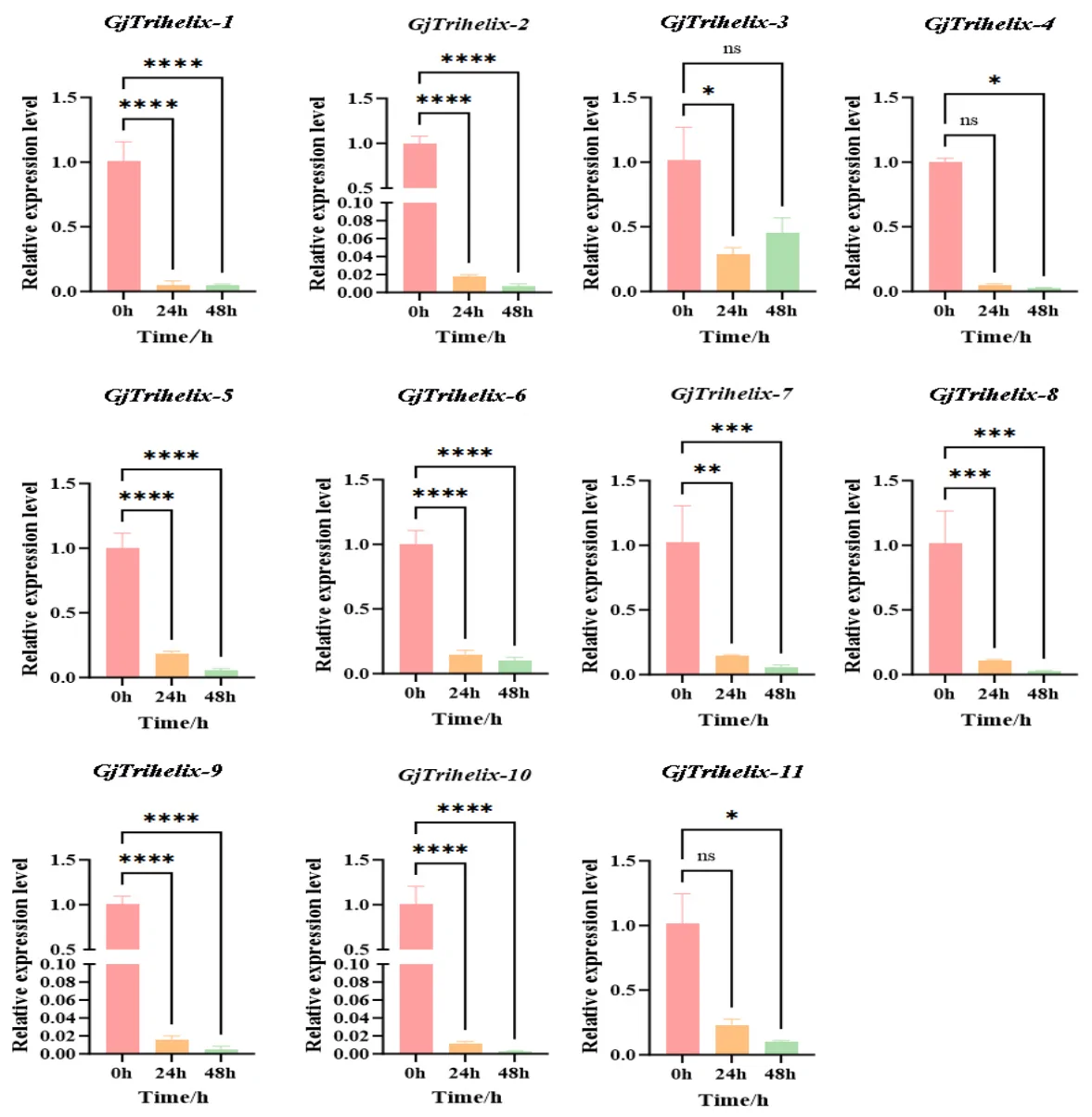
The expression patterns of *GjTrihelix* genes under ABA treatments. Note: qRT-PCR data were normalized using *18S* as the reference gene. Results are expressed as mean ± SD (*n* = 3). The * indicates significant differences compared to 0 h, as determined by *t*-test (* *p* < 0.05, ** *p* < 0.01, *** *p* < 0.001, **** *p* < 0.0001), ns represents no significant difference. Error bars represent the SD of three independent replicates.

To characterize the response pattern of the *GjTrihelix* gene family to ABA signaling, we determined the transcript abundance of 11 *GjTrihelix* members following ABA treatment by qRT-PCR. The results revealed that all genes except *GjTrihelix-3* exhibited a continuous down-regulation trend (Figure 10). Compared with the control, the transcript levels of these 10 genes decreased extremely significantly at 24 h after ABA treatment and were further reduced to an extremely low level at 48 h. Among them, *GjTrihelix-9* showed the most drastic reduction, with its expression declining to only 0.002-fold of the control at 48 h. *GjTrihelix-10* ranked second, with expression dropping to 0.004-fold, followed by *GjTrihelix-2* at 0.007-fold. Meanwhile, *GjTrihelix-4*, *GjTrihelix-7* and *GjTrihelix-8* were down-regulated to 0.030-fold, 0.047-fold and 0.031-fold of the control, respectively. These data demonstrated that the transcription of these genes was strongly repressed by ABA. Only *GjTrihelix-3* displayed a distinct expression profile. Its expression significantly decreased to 0.27-fold of the control at 24 h after ABA treatment but then markedly recovered to 0.52-fold at 48 h, exhibiting a “down-then-up” expression trend. This unique pattern suggests that *GjTrihelix-3* may play a distinctive role in ABA signal transduction. Collectively, these findings indicate that the majority of *GjTrihelix* genes are transcriptionally suppressed by exogenous ABA; only *GjTrihelix-3* showed a rebound in expression at the later stage of stress.

**Figure 11:**
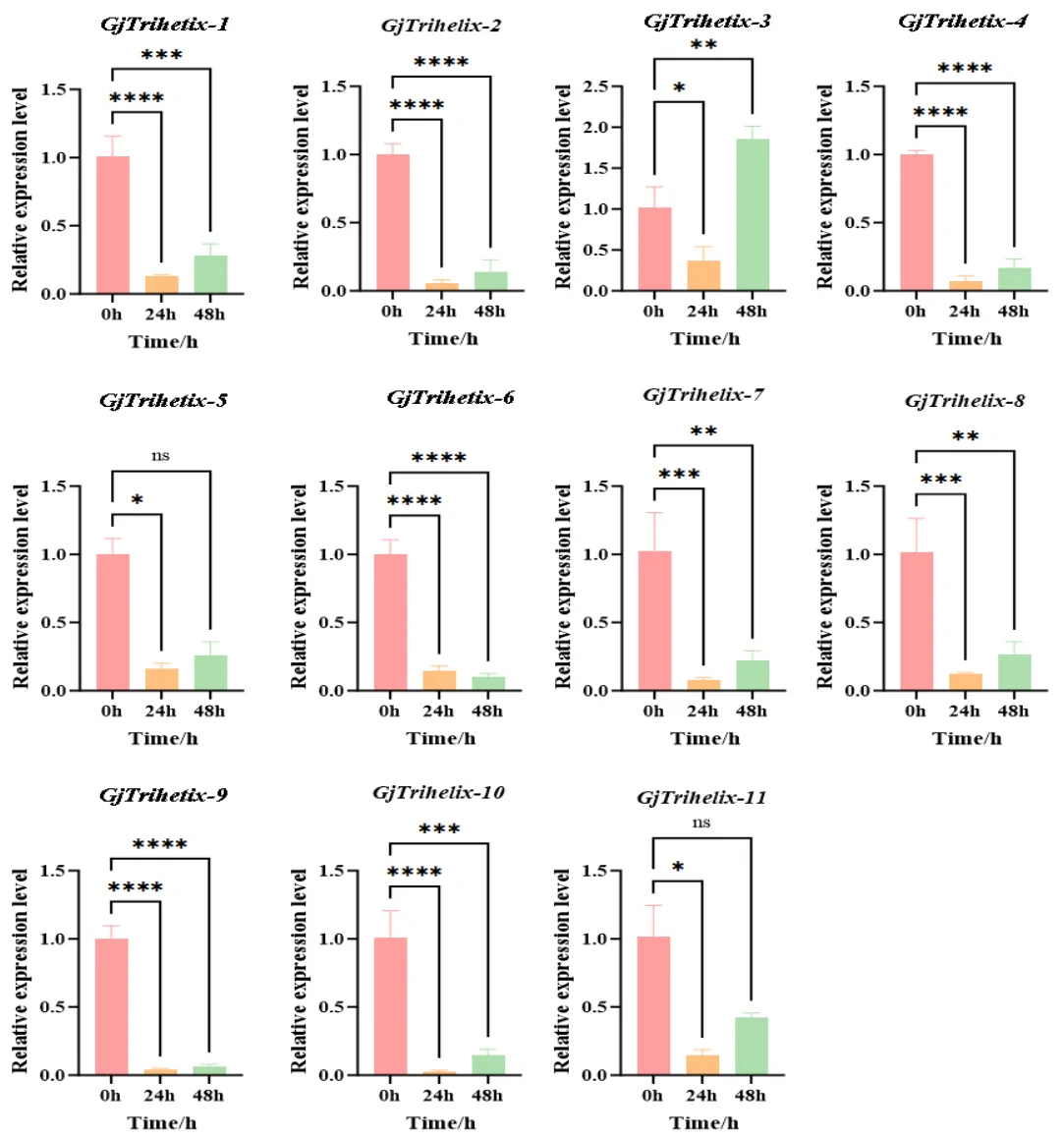
The expression patterns of *GjTrihelix* genes under GA_3_ treatments. Note: qRT-PCR data were normalized using *18S* as the reference gene. Results are expressed as mean ± SD (*n* = 3). The * indicates significant differences compared to 0 h, as determined by *t*-test (* *p* < 0.05, ** *p* < 0.01, *** *p* < 0.001, **** *p* < 0.0001), ns represents no significant difference. Error bars represent the SD of three independent replicates.

To investigate the response patterns of the *GjTrihelix* gene family to GA_3_, the relative expression levels of 11 *GjTrihelix* genes were examined by qRT-PCR at 0, 24, and 48 h following GA_3_ treatment. The results showed that all *GjTrihelix* genes responded to GA_3_ treatment to varying degrees, following an overall trend of initial transcriptional repression followed by recovery or activation (Figure 11). The transcript abundance of all 11 *GjTrihelix* genes decreased significantly at 24 h after GA_3_ treatment. Among them, *GjTrihelix-6*, *GjTrihelix-9* and *GjTrihelix-10* displayed the most dramatic reduction, with expression levels dropping to 0.07-fold, 0.04-fold and 0.02-fold of the control at 24 h, respectively. These data indicate that GA_3_ treatment exerts a widespread inhibitory effect on the transcription of the *GjTrihelix* gene family in the early stage. At 48 h, the expression of most genes rebounded to different extents. *GjTrihelix-3* exhibited the most striking change; its expression level reached 1.97-fold of the control at 48 h, showing a pattern of initial suppression followed by remarkable up-regulation. This implies that this gene may serve as a specific activator during the later phase of GA_3_ signal transduction. In addition, the expression levels of *GjTrihelix-1*, *GjTrihelix-5*, and *GjTrihelix-11* recovered to 0.28-fold, 0.26-fold, and 0.42-fold of the control, respectively, at 48 h, indicating a more sustained inhibitory effect of GA_3_ on these genes. Taken together, the transcription of the vast majority of *GjTrihelix* genes was first repressed and then partially restored under GA_3_ treatment. Notably, only *GjTrihelix-3* was markedly activated at the later stage of treatment, indicating that it functions as a unique positive regulator in the gibberellin signaling pathway.

**Figure 12:**
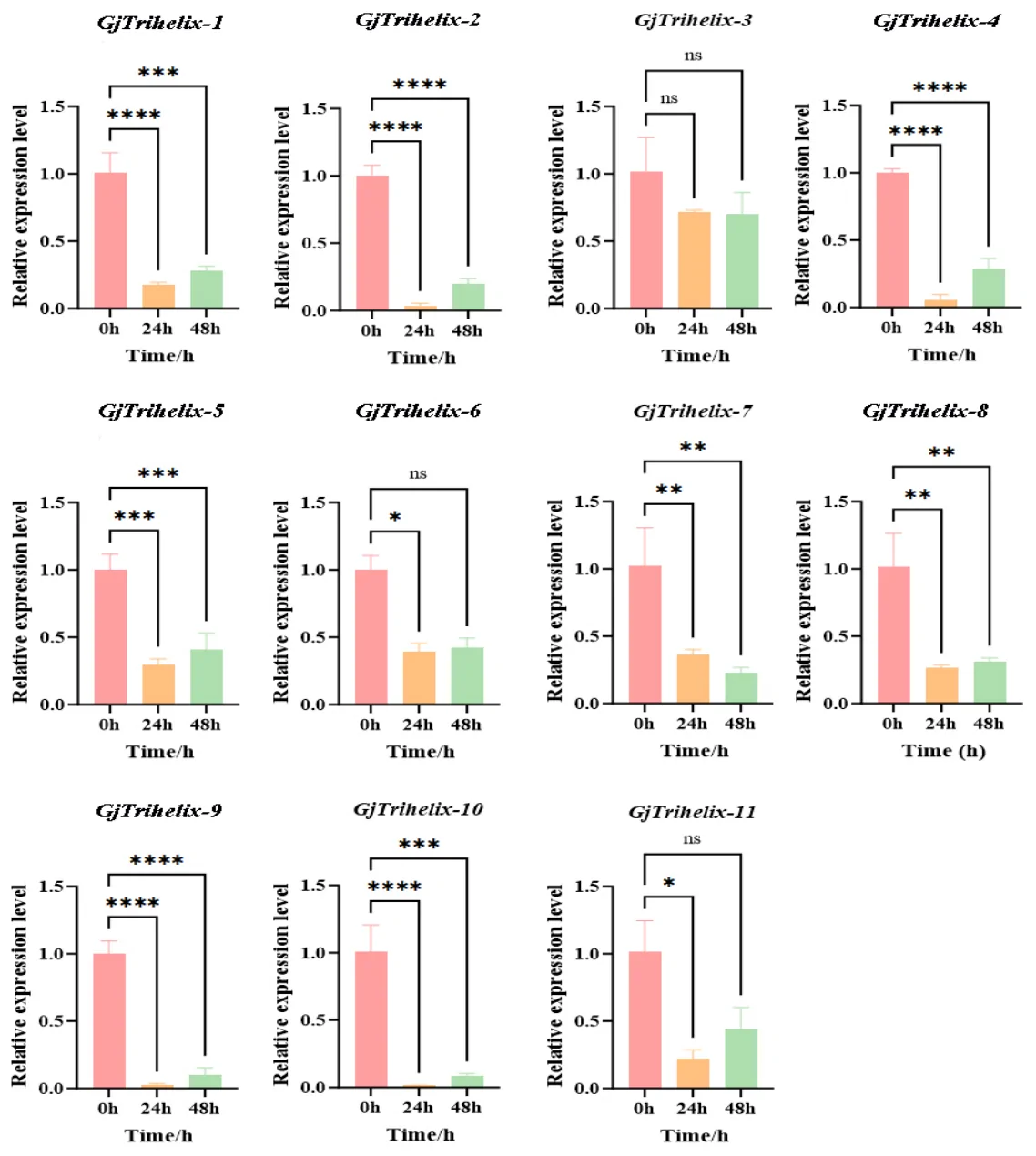
The expression patterns of *GjTrihelix* genes under IAA treatments. Note: qRT-PCR data were normalized using *18S* as the reference gene. Results are expressed as means ± SD (*n* = 3). The * indicates significant differences compared to 0 h, as determined by *t*-test (* *p* < 0.05, ** *p* < 0.01, *** *p* < 0.001, **** *p* < 0.0001), ns represents no significant difference. Error bars represent SD of three independent replicates.

Based on qRT-PCR data, the relative expression levels of 11 *GjTrihelix* genes were analyzed at 0, 24, and 48 h following IAA treatment (Figure 12). The results revealed that different genes exhibited markedly distinct response patterns to IAA, with an overall trend of initial suppression followed by partial recovery, suggesting functional divergence among family members in auxin signaling pathways. At 24 h of IAA treatment, obvious transcriptional suppression was observed in all 10 genes except *GjTrihelix-3*. Among them, *GjTrihelix-9* and *GjTrihelix-10* were most drastically inhibited. Their expression levels decreased to approximately 0.03-fold and 0.02-fold of the control at 24 h, and only rebounded slightly to 0.098-fold and 0.088-fold of the control at 48 h. *GjTrihelix-7* was persistently repressed by IAA, with its transcript abundance reduced to 0.36-fold at 24 h and 0.23-fold at 48 h relative to the untreated control. Notably, most genes, including *GjTrihelix-1/2/4/5/8/9/10/11*, showed varying degrees of recovery at 48 h, with *GjTrihelix-11* exhibiting the most pronounced recovery, reaching 0.44-fold of the control. In contrast, *GjTrihelix-3* maintained relatively stable expression under IAA treatment, with transcript levels at 0.72-fold and 0.70-fold of the control at 24 h and 48 h, respectively. Collectively, these findings indicate that the majority of *GjTrihelix* genes are initially subjected to strong transcriptional repression by exogenous IAA, while *GjTrihelix-3* remains largely unaffected, *GjTrihelix-7* is continuously repressed, and *GjTrihelix-9* and *GjTrihelix-10* are the most sensitive members. These results suggest that the *GjTrihelix* gene family may participate in the IAA signaling pathway through diversified expression regulation strategies.

### 3.12. Interaction Network and GO Function Analysis of G. jasminoides Trihelix Gene Family

**Figure 13:**
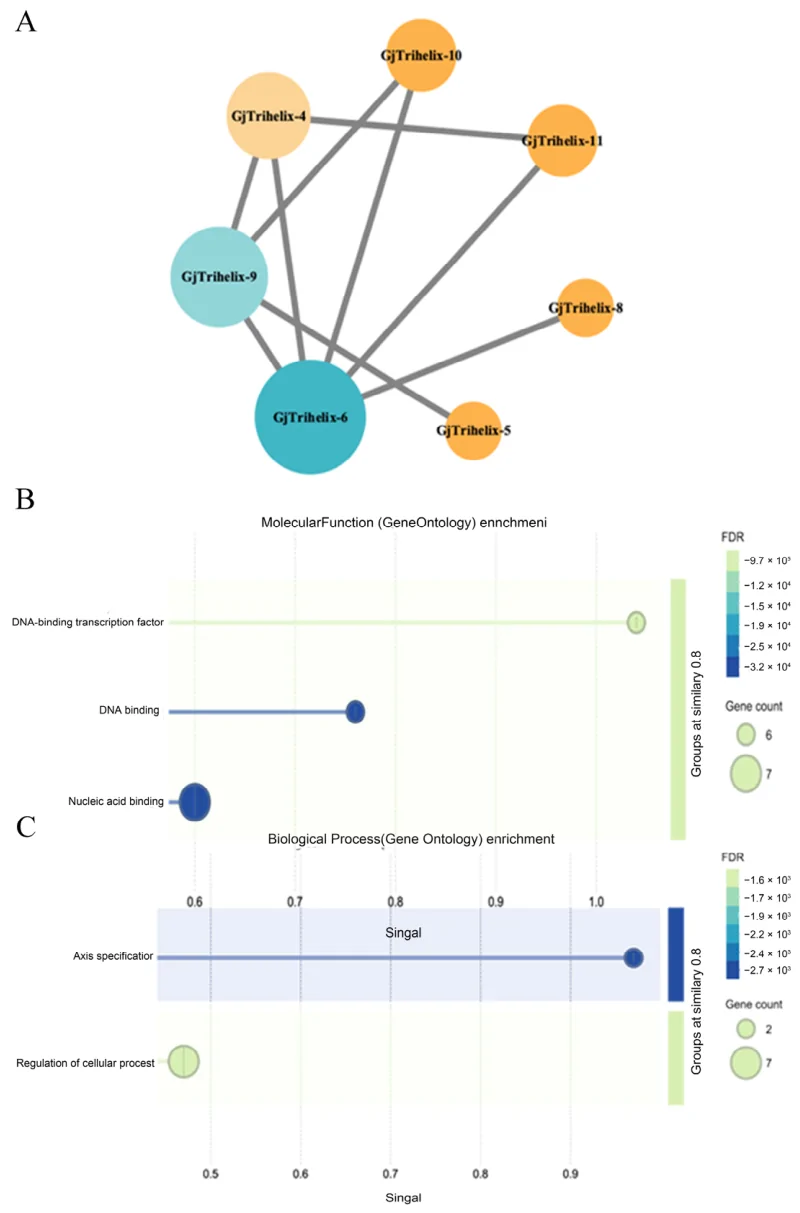
Interaction network and GO function of the Trihelix gene family of *G. jasminoides*. Note: (**A**) Protein–protein interaction analysis of GjTrihelix proteins. (**B**) GO Molecular Function (MF) annotation map. (**C**) GO Biological Process (BP) categories.

To dissect the functional interaction and potential regulatory roles of GjTrihelix proteins, a protein interaction network and Gene Ontology (GO) enrichment analysis were performed. The network revealed that GjTrihelix-6 acts as the central hub with the highest number of interactions (Figure 13), directly connecting to five other GjTrihelix proteins, and it is therefore considered a predicted candidate regulatory gene in this network. Secondly, GjTrihelix-9 showed four interacting partners. In contrast, GjTrihelix-8 exhibited the fewest interactions, with only GjTrihelix-6. To more comprehensively delineate the potential functions of Trihelix genes in *G. jasminoides*, detailed functional annotation and classification were performed using the EggNOG 5.0 tool (Figure 13B). In terms of molecular functions, *GjTrihelix* primarily exhibited binding activity and transcription factor activity, among others. Functional enrichment analysis revealed that GjTrihelix is potentially involved in a wide range of biological processes, including the regulation of cellular processes, as well as DNA-binding transcription factor activity and DNA and nucleic acid binding. Nevertheless, further experimental validation is required to elucidate their exact biological roles.

## 4. Discussion

*G. jasminoides* serves as a common Chinese herbal medicine and finds applications in the pharmaceutical, food, cosmetics, and dye industries. Both biotic and abiotic stresses severely limit plant growth productivity. To cope with these challenges, plants have developed sophisticated regulatory mechanisms, including transcriptional regulation mediated by TFs, which play a crucial role in coordinating gene expression in response to environmental stimuli [33,34]. Trihelix transcription factors are unique plant-specific regulatory proteins that rely on conserved triple-helix structural domains to bind GT cis-elements in target gene promoters, widely participating in plant growth, organ development, secondary metabolism and signal responses to hormones and stresses. At present, genome-wide identification of the Trihelix gene family has been completed across plant species. For instance, 56 *Trihelix* genes have been characterized in *Pinus massoniana* [34], 40 in *Sorghum bicolor* [35], 35 in potato [36], 36 in chrysanthemum [37], 31 in *Vitis amurensis* [38], 31 in rice [39], 30 in *Arabidopsis* [10], 29 in *Cucumis sativus* [40] and 28 in tomato [5]. In this study, 11 *GjTrihelix* genes were identified from the *G. jasminoides* genome, which were designated *GjTrihelix-1*-*GjTrihelix-11* according to their coding sequences and chromosomal physical positions. Phylogenetic analysis classified the Trihelix gene family into four distinct subfamilies: GT-1, GT-2, SIP1 and SH4. Furthermore, integrative bioinformatic profiling combined with transcriptomic data and qRT-PCR experimental validation systematically elucidated the evolutionary traits, structural divergence and versatile regulatory roles of these *GjTrihelix* members. This study establishes a fundamental theoretical framework for uncovering the molecular regulatory networks mediated by Trihelix transcription factors during fruit development, pigment biosynthesis and abiotic stress adaptation in *G. jasminoides*.

Homologous genes generally share identical or analogous biological functions. The biological roles of Trihelix transcription factors have been extensively characterized in model plants including *Arabidopsis*, rice, and tomato in previous studies [5,10]. The phylogenetic clustering patterns of GjTrihelix proteins, combined with interspecific collinearity relationships between *G. jasminoides* and *Arabidopsis*, rice, and soybean, provide robust clues for predicting the potential biological functions of *GjTrihelix* family members (Figure 1). In soybean, overexpression of GmGT-2F elevates seed oil content and increases the proportion of oleic and linoleic acids in fatty acid composition, whereas its knockout significantly reduces α-galactosidase activity [41]. Two rice OsGTγ subfamily members in rice, *OsGTγ-1* and *OsGTγ-2*, act as positive regulators that mediate salt stress tolerance [20,42]. In *Arabidopsis*, the GT-2 member AtGT2L interacts with calcium/calmodulin complexes to coordinate plant tolerance to cold and saline conditions [18]. In addition, AtGT-4 (GT-1 subfamily) forms a synergistic regulatory module with the AP2/ERF transcription factor TEM2 to activate the salt-responsive marker gene Cor15A, thereby reinforcing salt resistance [19]. Notably, GjTrihelix-8 clustered with ASIL1 in the phylogenetic tree. Functional assays have confirmed that ASIL1 suppresses the premature expression of LEC2, FUS3, ABI3, and seed storage protein genes, controlling seed filling duration [16]. Thus, we speculate these *GjTrihelix* genes may have the same functions and be related to stress responses. Interestingly, various plant hormones and stress-response-related elements were found in the promoters of the *GjTrihelix* genes mentioned above (Figure 3), further supporting our speculation that these *GjTrihelix* genes might be involved in stress regulation. However, the concrete function remains to be validated by further functional experiments.

Previous studies have shown that the GT gene family is involved in various growth and development processes such as root development and fruit maturation. The expression patterns of *Trihelix* genes during the growth and development of different tissues revealed that members of the *Trihelix* gene family exhibit certain tissue-specific expression patterns. For example, among the 11 *GjTrihelix* genes, 10 of them have the highest expression levels in the leaves, while *GjTrihelix-11* exhibited predominant expression in flowers, which is consistent with previous findings. Notably, the expression of eggplant SmGT2 is highly expressed in leaves; *SmGT3*, *SmGT6*, and *SmGT10* are highly expressed in the flowers [43]. In tomatoes, *SlGT-33* is predominantly expressed in mature leaves and stems [44]. In cucumber, *CsaTri1* and *CsaTri5* exhibit high expression levels in leaves, and *CsaTri12* shows elevated expression in flowers and fruits [40]. In potato, *StTrihelix32* shows high expression levels in flowers, petioles, and stems; *StTrihelix4* is highly expressed in stamens; and *StTrihelix5* and *StTrihelix13* is highly expressed in roots [36]. This clearly indicates that trihelix family genes exhibit tissue-specific expression patterns in *G. jasminoides*.

The *Trihelix* genes have been shown to not only play critical roles in plant morphogenesis, including trichome development, sepal formation, floral organ differentiation, stomatal patterning, embryogenesis, seed development, and abscission zone formation [5,45,46], but also to mediate plant defenses against biotic stress (pathogen invasion) and multiple abiotic stimuli, including cold, drought, and high salinity, as well as phytohormone signaling cascades [38]. For example, members of the *GjTrihelix* family exhibit significant functional differences in their responses to the melatonin signal. Among them, five *GjTrihelix* genes were significantly induced by melatonin, whereas another five displayed repressed transcript accumulation under the same treatment. Intriguingly, *GjTrihelix-4* exhibited no detectable transcriptional response to melatonin supplementation. In pear, *PbrGT15* positively regulates resistance to black spot disease caused by *Alternaria alternata* (Fr.) Keissl. Silencing *PbrGT15* significantly increases susceptibility [47,48]. Moreover, *ZmGT-3b* expression declines upon *Fusarium graminearum* infection. It interacts with *ZmHY5* to coordinate metabolic trade-offs between defense and growth, balancing disease resistance and development [49]. Under salt stress, 10 out of the 11 *GjTrihelix* genes displayed progressively reduced transcript abundance over the treatment time course. Among them, *GjTrihelix-9* and *GjTrihelix-10* underwent the most dramatic transcriptional suppression: their expression levels fell to only 0.007-fold and 0.0008-fold of the control at 48 h post salt exposure, respectively, reflecting nearly complete transcriptional shutdown. In contrast, *GjTrihelix-3* presented a unique biphasic expression profile, with transcript levels initially declining followed by a subsequent rebound. Accumulating studies have verified that the *Arabidopsis Trihelix* gene *AtGT2L* is significantly induced by salt, cold, and abscisic acid (ABA) treatments. CRISPR/Cas9-mediated knockout of the *SlGT30* in tomato reduced stomatal density and water loss rate, enhancing drought resistance [50]. In rice, *OsGTγ-2* positively regulates salt tolerance, as its overexpression improves salt resistance while CRISPR-mediated knockout leads to salt hypersensitivity [42]. Similarly, heterologous expression of cotton *GhGT26* confers enhanced salt tolerance in transgenic *Arabidopsis* [51]. In apple, the expression levels of *MdTH4* and *MdTH24* are markedly modulated by soil salinity [52].

In our study, many common cis-acting elements, such as ARE, LTR, MBS, and TC-rich repeats, were identified in the promoter region of *GjTrihelix*. These *GjTrihelix* genes contain at least hormone-acting elements, which indicates that the expression level of these genes will change under the action of hormones (Figure 3). To deeply understand the hormone response mechanism of the *GjTrihelix* genes, qRT-PCR experiments were conducted under different hormone treatments, such as ABA, GA3 and IAA, in leaves (Figure 10, Figure 11 and Figure 12). In this study, exogenous ABA, GA3, and IAA treatments markedly suppressed the transcription of nearly all *GjTrihelix* genes. Under ABA treatment, the transcript abundance of *GjTrihelix-9* plummeted to merely 0.002-fold relative to the control at 48 h, followed by *GjTrihelix-10*, whose expression decreased to 0.004-fold of the control level. Upon IAA exposure, the expression of *GjTrihelix-9* and *GjTrihelix-10* fell to approximately 0.03-fold and 0.02-fold of the control at 24 h, respectively, and only slightly recovered to 0.098-fold and 0.088-fold at 48 h. Consistent with this inhibitory trend observed in *G. jasminoides*, similar expression patterns were also documented in *P. massoniana*. Specifically, seven out of eight PmGT family members were significantly suppressed by ABA, with only *PmGT38* exempted from this inhibitory effect. Under IAA treatment, PmGT14 exhibited induced expression at the 12 h time point, whereas all other *PmGT* genes were transcriptionally repressed. Moreover, every *PmGT* gene displayed prominent down-regulation in response to GA3 application [34]. Nevertheless, distinct regulatory responses of *Trihelix* members to phytohormones have been reported in other plant species, revealing interspecific functional divergence. For instance, rice *OsGTγ-1* is up-regulated by ABA, implying its vital participation in ABA-mediated stress signaling pathways. Likewise, *Arabidopsis AtGT2L* can be activated by both salt stress and ABA exposure [18]. Another tomato *Trihelix* gene, *SlGT-33*, which falls into the SIP1 clade, is predominantly expressed in mature leaves and stems. Its transcription is activated by ABA and dehydration but repressed by GA and low temperature. Functional assays further demonstrated that over-expressing *SlGT-33* inhibits chlorophyll biosynthesis yet facilitates fruit setting relative to wild-type plants under sustained cold stress [44]. In eggplant, *SmGT13* and *SmGT12* were found to be involved in the cross-regulation of ABA and SA, respectively [43]. Collectively, these divergent expression profiles in response to various hormones and stresses strongly suggest that the Trihelix gene family has experienced substantial functional diversification during plant evolution. Nevertheless, such transcript-level correlations cannot establish causal biological functions. Further genetic functional assays, including stable genetic transformation, are therefore required in future work to verify the biological roles of these Trihelix genes and to dissect the regulatory networks of their downstream target genes.

## 5. Conclusions

In this study, a genome-wide identification and systematic characterization of the *Trihelix* gene family were performed in *G. jasminoides*. A total of 11 *GjTrihelix* members were identified, which were classified into four subfamilies (GT-1, GT-2, SIP and SH4) and unevenly distributed across five chromosomes. Notable divergences were observed in gene structures and motif compositions among members, with a segmental duplication event detected between *GjTrihelix-3* and *GjTrihelix-10*. Interspecific synteny analysis indicated the closest evolutionary relationship between *G. jasminoides* and soybean. Promoter cis-element analysis revealed that *GjTrihelix* genes are enriched in hormone-, stress-, and light-responsive elements. Expression profiling demonstrated distinct tissue-specific patterns, with *GjTrihelix-11* potentially playing a specialized role in flower development, while *GjTrihelix-1*, *-3*, *-6*, and *-10* may be involved in early fruit development, and *GjTrihelix-11* in pigment accumulation during fruit ripening. Furthermore, multiple *GjTrihelix* genes responded to melatonin treatment and *Botryosphaeria dothidea* infection, and most *GjTrihelix* genes were significantly down-regulated under NaCl, ABA, GA3 and IAA stresses. Protein–protein interaction network prediction identified *GjTrihelix-6* as a potential key regulatory node. Collectively, these findings provide a solid foundation for further functional studies of *Trihelix* genes in *G. jasminoides*.

## Figures and Tables

**Table 1:** Analysis of physical and chemical properties of Trihelix proteins from *G. jasminoides*.

Gene ID	Gene Name	Number of Amino Acids	Molecular Weight	Theoretical pI	Instability Index	Subcellular Localization	Grand Average of Hydropathicity
IMPTGJA1N27720_1	*GjTrihelix-1*	579	65,226.99	5.68	55.39	Nucleus	−0.946
IMPTGJA1N28316_1	*GjTrihelix-2*	288	33,474.53	8.32	57.87	Nucleus	−1.178
IMPTGJA1N31027_1	*GjTrihelix-3*	388	44,084.21	5.60	48.82	Nucleus	−0.807
IMPTGJA1N33021_1	*GjTrihelix-4*	585	66,029.46	6.42	51.20	Nucleus Peroxisome	−0.971
IMPTGJA1N55042_1	*GjTrihelix-5*	123	13,925.55	9.14	64.66	Nucleus	−1.160
IMPTGJA1N59694_1	*GjTrihelix-6*	606	68,379.08	6.34	56.52	Nucleus	−0.917
IMPTGJA1N53599_1	*GjTrihelix-7*	359	41,222.75	5.18	59.94	Nucleus	−1.133
IMPTGJA1N47726_1	*GjTrihelix-8*	667	73,058.28	6.35	58.08	Nucleus	−0.899
IMPTGJA1N24434_1	*GjTrihelix-9*	369	41,982.87	7.15	58.56	Nucleus	−0.992
IMPTGJA1N15413_1	*GjTrihelix-10*	401	45,526.52	5.39	54.26	Nucleus	−0.875
IMPTGJA1N33773_1	*GjTrihelix-11*	894	98,917.37	8.95	41.09	Chloroplast	−0.375

**Table 2:** Secondary structure analysis of *G. jasminoides* Trihelix proteins.

Protein Name	α-Helix		Extended Chain		Random Coil	
	Quantity	Proportion/%	Quantity	Proportion/%	Quantity	Proportion/%
GjTrihelix-1	195	33.68	2	0.35	382	65.98
GjTrihelix-2	157	54.51	0	0	131	45.49
GjTrihelix-3	101	26.03	35	9.02	252	64.95
GjTrihelix-4	222	37.95	0	0	357	61.03
GjTrihelix-5	60	48.78	0	0	63	51.22
GjTrihelix-6	197	32.51	2	0.33	407	67.16
GjTrihelix-7	156	43.45	0	0	203	56.55
GjTrihelix-8	206	30.88	5	0.75	456	68.37
GjTrihelix-9	143	38.75	4	1.08	222	60.16
GjTrihelix-10	102	25.44	28	6.98	271	67.58
GjTrihelix-11	276	30.87	143	16	475	53.13

## Data Availability

The original contributions presented in this study are included in the article/Supplementary Materials. Further inquiries can be directed to the corresponding authors.

## Supplementary Materials

The following supporting information can be downloaded at https://www.mdpi.com/article/10.3390/biom16091298/s1: Figure S1: the tertiary structure prediction of GjTrihelix proteins in *G. jasminoides*; Figure S2: Considering that Neighbor-Joining (NJ) has limitations in phylogenetic reconstruction, we have newly performed maximum-likelihood (ML) phylogenetic analysis with optimal amino-acid model selection using the full 160-protein; Figure S3: Melt Curve Plot0; Figure S4: Melt Curve PlotABA-24; Figure S5: Melt Curve PlotABA-48; Figure S6: Melt Curve Plotbud; Figure S7: Melt Curve PlotGA3-24; Figure S8: Melt Curve PlotGA3-48; Figure S9: Melt Curve PlotIAA-24; Figure S10: Melt Curve PlotIAA-48; Figure S11: Melt Curve Plotleaf; Figure S12: Melt Curve PlotNaCL-24; Figure S13: Melt Curve PlotNaCL-48; Figure S14: Melt Curve Plotsingleflower; Figure S15: Melt Curve Plotstem; Table S1: primer sequences of GjTrihelix genes; Table S2: Expression patterns of Trihelix genes in *G. jasminoides* peels at different developmental stages; Table S3: Expression patterns of *Trihelix* genes in *G. jasminoides* sarcocarp at different developmental stages; Table S4: Expression patterns of *G. jasminoides* Trihelix gene family in Melatonin treatment; Table S5: Expression patterns of the *G. jasminoides* Trihelix gene family under *Botryosphaeria dothidea* treatment; Table S6: Detailed information of the 11 *GjTrihelix* genes identified in *Gardenia jasminoides*; Table S7: The domain organization diagrams of GjTrihelix.

## References

[1] Chen L., Li M., Yang Z., Tao W., Wang P., Tian X., Li X., Wang W. (2020). *Gardenia jasminoides* Ellis: Ethnopharmacology, phytochemistry, and pharmacological and industrial applications of an important traditional chinese medicine. J. Ethnopharmacol..

[2] Zhang N., Bian Y., Yao L. (2022). Essential Oils of *Gardenia jasminoides* J. Ellis and *Gardenia jasminoides* f. longicarpa Z.W. Xie & M. Okada Flowers: Chemical characterization and assessment of anti-inflammatory effects in alveolar macrophage. Pharmaceutics.

[3] Chen Q.C., Youn U., Min B.-S., Bae K. (2008). Pyronane Monoterpenoids from the Fruit of *Gardenia jasminoides*. J. Nat. Prod..

[4] Zhou D. (1999). Regulatory mechanism of plant gene transcription by GT-elements and GT-factors. Trends Plant Sci..

[5] Yu C., Cai X., Ye Z., Li H. (2015). Genome-wide identification and expression profiling analysis of trihelix gene family in tomato. Biochem. Biophys. Res. Commun..

[6] Green P.J., Kay S.A., Chua N.H. (1987). sequence-specific interactions of a pea nuclear factor with light-responsive elements upstream of the *rbcS-3A* Gene. EMBO J..

[7] Dehesh K., Hung H., Tepperman J., Quail P. (1992). GT-2: A Transcription factor with twin autonomous dna-binding domains of closely related but different target sequence specificity. EMBO J..

[8] O’GRady K., Goekjian V.H., Nairn C.J., Nagao R.T., Key J.L. (2001). The transcript abundance of GmGT-2, a new member of the GT-2 family of transcription factors from soybean, is down-regulated by light in a phytochrome-dependent manner. Plant Mol. Biol..

[9] Nakamura K., Sato Y., Yamaguchi T., Hirano H.Y. (2012). The SH4 gene encoding a trihelix transcription factor is involved in regulating the grain shattering trait in rice. Plant Cell Physiol..

[10] Kaplan-Levy R.N., Brewer P.B., Quon T., Smyth D.R. (2012). The trihelix family of transcription factors–light, stress and development. Trends Plant Sci..

[11] Cheng X., Xiong R., Yan H., Gao Y., Liu H., Wu M., Xiang Y. (2019). The Trihelix family of transcription factors: Functional and evolutionary analysis in moso bamboo (*Phyllostachys edulis*). BMC Plant Biol..

[12] Yang L., Qi S., Touqeer A., Li H., Zhang X., Liu X., Wu S. (2020). *SlGT11* controls floral organ patterning and floral determinacy in tomato. BMC Plant Biol..

[13] Weng H., Yoo C.Y., Gosney M.J., Hasegawa P.M., Mickelbart M.V. (2012). Poplar GTL1 is a Ca^2+^/calmodulin-binding transcription factor that functions in plant water use efficiency and drought tolerance. PLoS ONE.

[14] Yoo C.Y., Pence H.E., Jin J.B., Miura K., Gosney M.J., Hasegawa P.M., Mickelbart M.V. (2010). The *Arabidopsis* GTL1 transcription factor regulates water use efficiency and drought tolerance by modulating stomatal density via transrepression of *SDD1*. Plant Cell.

[15] Tzafrir I., Pena-Muralla R., Dickerman A., Berg M., Rogers R., Hutchens S., Sweeney T.C., McElver J., Aux G., Patton D. (2004). Identification of genes required for embryo development in *Arabidopsis*. Plant Physiol..

[16] Gao M.J., Li X., Lui H., Gropp G.M., Lydiate D.D., Wei S., Hegedus D.D. (2011). ASIL1 is required for proper timing of seed filling in *Arabidopsis*. Plant Signal. Behav..

[17] Yu C., Song L., Song J., Ouyang B., Guo L., Shang L., Wang T., Li H., Zhang J., Ye Z. (2018). ShCIGT, a Trihelix family gene, mediates cold and drought tolerance by interacting with *SnRK1* in tomato. Plant Sci..

[18] Xi J., Qiu Y., Du L., Poovaiah B. (2012). Plant-specific trihelix transcription factor AtGT2L interacts with calcium/calmodulin and responds to cold and salt stresses. Plant Sci..

[19] Wang X.-H., Li Q.-T., Chen H.-W., Zhang W.-K., Ma B., Chen S.-Y., Zhang J.-S. (2014). Trihelix transcription factor GT-4 mediates salt tolerance via interaction with TEM2 in *Arabidopsis*. BMC Plant Biol..

[20] Fang Y., Xie K., Hou X., Hu H., Xiong L. (2009). Systematic analysis of GT factor family of rice reveals a novel subfamily involved in stress sesponses. Mol. Genet. Genom..

[21] Luo J., Tang S., Mei F., Peng X., Li J., Li X., Yan X., Zeng X., Liu F., Wu Y. (2017). BnSIP1-1, a trihelix family gene, mediates abiotic stress tolerance and ABA signaling in *Brassica napus*. Front. Plant Sci..

[22] Sun W., Chen Y., Yao M., Zhan J., Chen H., Zhao G., Zou L., Xiang D., Wu X., Wan Y. (2024). Genome-wide characterization of *trihelix* genes reveals *Cqtrihelix23* enhances the salt tolerance in quinoa (*Chenopodium quinoa*). Physiol. Plant..

[23] Cao Y., Cheng Z., Sun X., Zhu M., Yue L., Liu H., Wu X., Zhang J., Duan C. (2024). Genome-wide identification of the trihelix transcription factor family and functional analysis of *ZmTHX15* in Maize. Int. J. Mol. Sci..

[24] Fu M., Li F., Zhou S., Guo P., Chen Y., Xie Q., Chen G., Hu Z. (2023). Trihelix transcription factor slgt31 regulates fruit ripening mediated by ethylene in tomato. J. Exp. Bot..

[25] Quevillon E., Silventoinen V., Pillai S., Harte N., Mulder N., Apweiler R., Lopez R. (2005). InterProScan: Protein domains identifier. Nucleic Acids Res..

[26] Letunic I., Doerks T., Bork P. (2008). SMART 6: Recent updates and new developments. Nucleic Acids Res..

[27] Gasteiger E., Gattiker A., Hoogland C., Ivanyi I., Appel R.D., Bairoch A. (2003). ExPASy: The proteomics server for in-depth protein knowledge and analysis. Nucleic Acids Res..

[28] Waterhouse A., Bertoni M., Bienert S., Studer G., Tauriello G., Gumienny R., Heer F.T., De Beer T.A.P., Rempfer C., Bordoli L. (2018). SWISS-MODEL: Homology modelling of protein structures and complexes. Nucleic Acids Res..

[29] Chen C., Wu Y., Li J., Wang X., Zeng Z., Xu J., Liu Y., Feng J., Chen H., He Y. (2023). TBtools-II: A “one for all, all for one” bioinformatics platform for biological big-data mining. Mol. Plant.

[30] Liu Q., Gu Z., Fu C., Yang C. (2021). Cloning and expression pattern analysis of UGT gene family *UGT86A1* and *UGT85A2* in *Gardenia jasminoides*. J. Cent. South Univ. For. Technol..

[31] Kohl M., Wiese S., Warscheid B. (2011). Cytoscape: Software for visualization and analysis of biological networks. Methods Mol. Biol..

[32] Altaf M.A., Shahid R., Ren M.-X., Naz S., Altaf M.M., Khan L.U., Tiwari R.K., Lal M.K., Shahid M.A., Kumar R. (2022). Melatonin improves drought stress tolerance of tomato by modulating plant growth, root architecture, photosynthesis, and antioxidant defense system. Antioxidants.

[33] Tan L., Rasool A., Almutairy L.A., Azeem F., Jehan I., Masroor A., Attia K.A., Fiaz S., Shah A.A. (2025). Trihelix transcription factors are involved in drought stress response of *Mangifera indica*. Mol. Biol. Rep..

[34] Liu P., Wang D., Xu S., Sun S., Yang M., Chen M., Ji K. (2025). Genome-wide identification and characterization of the trihelix transcription factor family in *Pinus massoniana* and gene expression patterns analysis. Plants.

[35] Li K., Duan L., Zhang Y., Shi M., Chen S., Yang M., Ding Y., Peng Y., Dong Y., Yang H. (2021). Genome-wide identification and expression profile analysis of trihelix transcription factor family genes in response to abiotic stress in sorghum [*Sorghum bicolor* (L.) Moench]. BMC Genom..

[36] Yang H., Wang Y., Liu T., Yao W., Fan X., Yu B., Shi G. (2025). Genome-wide identification of potato Trihelix gene family and its response to different abiotic stresses. BMC Plant Biol..

[37] Song A., Wu D., Fan Q., Tian C., Chen S., Guan Z., Xin J., Zhao K., Chen F. (2016). Transcriptome-wide identification and expression profiling analysis of *Chrysanthemum* Trihelix transcription factors. Int. J. Mol. Sci..

[38] SHuang X., Lu S., Feng Y., Shi G., Liang G., Mao J. (2025). Genome-wide identification and characterization of the Trihelix transcription factor family and functional analysis of *VaTrihelix23* under low temperatures stress in *Vitis amurensis*. BMC Plant Biol..

[39] Ji J.-H., Zhou Y.-J., Wu H.-H., Yang L.-M. (2015). Genome-wide analysis and functional prediction of the Trihelix transcription factor family in rice. Yi Chuan.

[40] Xie T., Xue X., Chen L., Wang Y., Geng X. (2025). Genome-wide identification and expression analysis of trihelix transcription factor family in cucumber (*Cucumis sativus* L.) and their roles in biotic stress responses. BMC Genom..

[41] Li S., Cui Y., Liu Y., Yang M., Wu X., Chen Q., Zhao Y. (2026). The GmGT-2F, a trihelix transcription factor, regulates seed oil content by directly activating GmAGAL transcription in soybean. J. Integr. Plant Biol..

[42] Liu X., Wu D., Shan T., Xu S., Qin R., Li H., Negm M., Wu D., Li J. (2020). The trihelix transcription factor OsGTγ-2 is involved adaption to salt stress in rice. Plant Mol. Biol..

[43] Lan Y., Gong F., Li C., Xia F., Li Y., Liu X., Liu D., Liang G., Fang C., Cai P. (2024). New insights into the evolution analysis of trihelix gene family in eggplant (*Solanum melongena* L.) and expression analysis under abiotic stress. BMC Genom..

[44] Zhu Z., Liu X., Li Z., Ye J., Zhong Z., Fu S., Yu M., Bai J., Cui B. (2025). A *SIP1* gene of Trihelix family, SlGT-33, promotes chilling tolerance and fruits set under prolonged cold stress in tomatoes. BMC Plant Biol..

[45] Du H., Huang M., Liu L. (2015). The genome wide analysis of gt ttranscription factors that respond to drought and waterlogging stresses in Maize. Euphytica.

[46] Osorio M.B., Bücker-Neto L., Castilhos G., Turchetto-Zolet A.C., Wiebke-Strohm B., Bodanese-Zanettini M.H., Margis-Pinheiro M. (2012). Identification and in silico characterization of soybean trihelix-GT and bHLH transcription factors involved in stress responses. Genet. Mol. Biol..

[47] Zhang C., Yao X., Zhang Y., Zhao S., Liu J., Wu G., Yan X., Luo J. (2023). Transcriptomic profiling highlights the ABA response role of *BnSIP1-1* in *Brassica napus*. Int. J. Mol. Sci..

[48] Zhang Z., Xie H., Xu X., Chen X., Li T., Huang X., Zhang S. (2023). Genome-wide identification of trihelix transcription factor family genes in pear (*Pyrus bretschneideri*) and functional characterization of *PbrGT15* in black spot resistance. Hortic. Adv..

[49] Zhang Q., Zhong T., E L., Xu M., Dai W., Sun S., Ye J. (2021). GT factor ZmGT-3b is associated with regulation of photosynthesis and defense response to *Fusarium graminearum* infection in maize seedling. Front. Plant Sci..

[50] Lv H.M., Wang X.W., Dong X.N., Gao M., Dong D.H., Li C.H., Jing S.R., Guo Y.D., Zhang N. (2025). CRISPR/Cas9 edited *SlGT30* improved both drought resistance and fruit yield through endoreduplication. Plant Cell Environ..

[51] Li Y., Hu Z., Dong Y., Xie Z. (2022). Trihelix Transcriptional factor GhGT26 of cotton enhances salinity tolerance in *Arabidopsis*. Plants.

[52] Kuzmitskaya P., Koroleva E., Urbanovich O. (2023). Genome-wide identification of trihelix transcription factors in the apple genome in silico. J. Appl. Genet..

