# Supplementary material for: The Trihelix Genes in Gardenia jasminoides Evolution, Expression Profiles, and Potential Regulatory Functions in Growth, Development, and Multiple Stress Responses
Source: Biomolecules. 2026 Sep 8;16(9):1298. doi: 10.3390/biom16091298 (PMC13604084; doi:10.3390/biom16091298)
Supplement: Supplementary file 1 [file biomolecules-16-01298-s001.zip › biomolecules-4502881-supplementary.pdf]

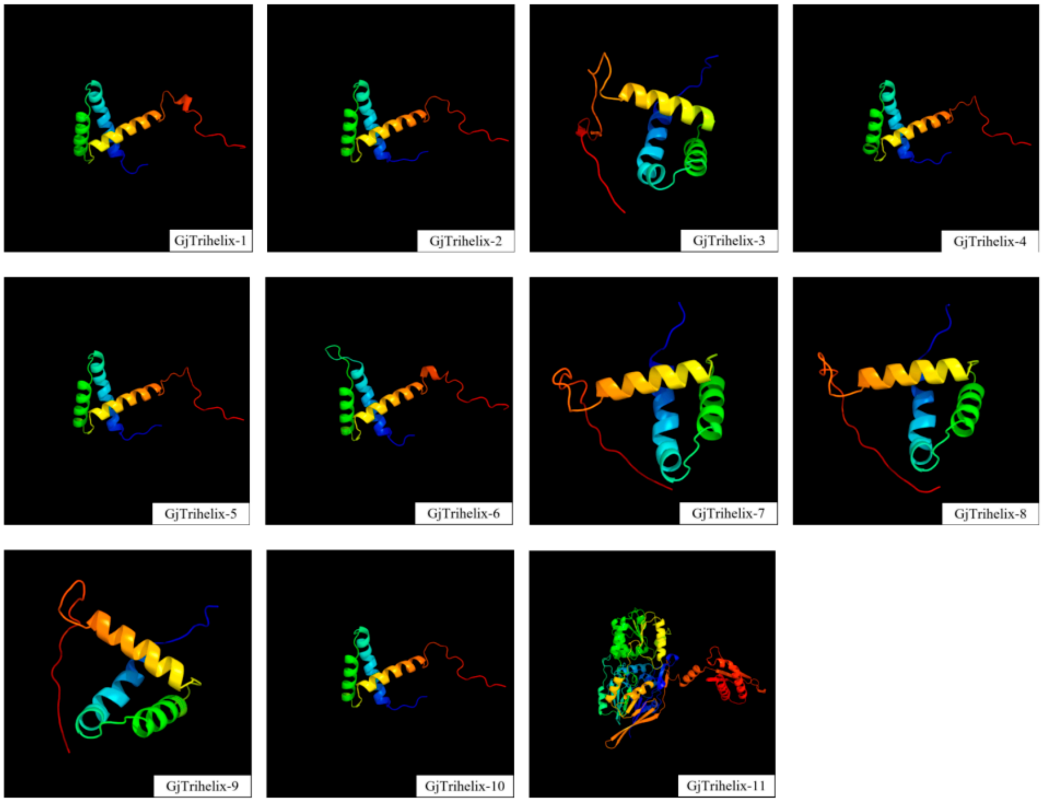

Figure S1. The tertiary structure prediction of GjTrihelix proteins in *G. jasminoides*

Table S1 Primer sequences of *GjTrihelix* genes

Gene name	Forward primer	Reverse primer
18S-RT	TCAACTTTCGATGGTAGGATAGTG	CCGTGTCAGGATTGGGTAATTT
GjTrihelix-1	CGCCTCTGCTGAAAACTTGG	CTTTGGAGCAGCCACCGTTA
GjTrihelix-2	GTTGCAAACAATTTTCGCGTCT	CCGCTTTAATCTTCCGTTTCTT
GjTrihelix-3	ATAGACGGAACTGCTGATGCC	CGATCCGACTCATTGTACAGACAT
GjTrihelix-4	CTCCGAACGGTGATGGTGAT	TTTTCCCACAGGTTCTCGGC
GjTrihelix-5	TGGATATCATCGAAGTGCCAAGA	TGCCGACCAGATCGACATTC
GjTrihelix-6	ACCTCCTCAGATACACTAGCCAT	CTAGGCCATCGCTTACTGGC
GjTrihelix-7	CATCAGGGAGGGAGTCAGAAG	ATGCTTCTTCCCTCGCCATTC
GjTrihelix-8	CATGGCACTGTTTCATCGGC	GCGGCAATTGTTGCGATTGA
GjTrihelix-9	CACCCCTACTCATCACCAGC	GCTTGGGGAAGTCATCGTCA
GjTrihelix-10	CCTGCCACCGGAGAAATGAT	AAGATAATGGGCGGGGTGTG
GjTrihelix-11	ACCCGAACCCTAGTAGGCAAT	TCCATGCTCTTTCCAGGTCC

Table S2. Expression patterns of Trihelix genes in *G. jasminoides* peels at different developmental stages.

Gene name	SG-1	SG-2	SG-3	SR-1	SR-2	SR-3
GjTrihelix-1	1.0975	1	0.52	0	0	0
GjTrihelix-2	2.9725	2.95	2.815	2.995	2.44	2.2825
GjTrihelix-3	15.0275	14.75	10.865	8.31	6.75	6.33
GjTrihelix-4	3.015	2.19	1.4625	1.8375	1.32	1.185

GjTrihelix-5	73.5725	52.91	51.0125	82.5075	79.05	73.155
GjTrihelix-6	25.89	23.79	22.0725	2.845	2.29	1.525
GjTrihelix-7	48.4175	47.66	46.0325	67.7775	65.4	56.856
GjTrihelix-8	40.395	39.27	37.815	55.96	38.11	34.225
GjTrihelix-9	4.285	3.64	2.8975	3.17	2.33	2.195
GjTrihelix-10	21.6925	15.46	11.92	16.2175	8.26	5.11
GjTrihelix-11	11.3725	10.03	9.7225	26.35	24.67	15.0925

Table S3. Expression patterns of *Trihelix* genes in *G. jasminoides* sarcocarp at different developmental stages.

Gene name	PG-1	PG-2	PG-3	PR-1	PR-2	PR-3
GjTrihelix-1	4.6375	3.91	2.905	0.4	0.16	0.13
GjTrihelix-2	4.645	3.7	2.9875	7.42	5.05	3.0625
GjTrihelix-3	21.1275	18.66	18.315	10.405	9.04	7.6075
GjTrihelix-4	2.8325	2.6	2.5025	0.875	0.8	0.5975
GjTrihelix-5	98.0025	87.51	85.11	103.585	77.8	62.47
GjTrihelix-6	39.5	21.32	18.08	9.7175	6.98	6.8675
GjTrihelix-7	65.3925	63.78	52.785	67.65	57.75	56.1375
GjTrihelix-8	40.56	34.59	33.6	40.11	36.51	30.54
GjTrihelix-9	3.9125	3.74	3.62	4.9625	4.07	4.01
GjTrihelix-10	20.84	17.21	14.4125	14.645	11.54	8.3525
GjTrihelix-11	15.1125	13.68	13.4625	18.7725	16.59	15.2175

Table S4 Expression patterns of *G. jasminoides* Trihelix gene family in Melatonin treatment

Gene name	Melatonin-1	Melatonin-2	Melatonin-3	Mock-1	Mock-2	Mock-3
GjTrihelix-1	0.3325	0.31	0.28	4.4	2.68	2.12
GjTrihelix-2	3.7375	3.7	2.8525	3.92	1.58	1.03
GjTrihelix-3	7.965	7.11	6.6825	23.69	21.42	20.07
GjTrihelix-4	0.075	0.06	0.0375	0	0.04	0
GjTrihelix-5	71.73	64.32	59.7975	52.56	50.72	46.19
GjTrihelix-6	4.2375	3.96	3.7125	17.43	10.73	9.46
GjTrihelix-7	100	95.54	80.8925	82.99	70.44	43.98
GjTrihelix-8	32.0275	29.56	26.6875	26.99	22.22	15.6
GjTrihelix-9	6.74	5.9	4.6325	4.45	4.12	3.76
GjTrihelix-10	8.21	7.61	6.155	23.67	17.35	16.32
GjTrihelix-11	11.6925	9.66	9.4725	61.16	39.14	38.86

Table S5 Expression patterns of the *G. jasminoides* Trihelix gene family under *Botryosphaeria dothidea* treatment

Gene name	0h-1	0h-2	0h-3	12h-1	12h-2	12h-3	24h-1	24h-2	24h-3	48h-1	48h-2	48h-3	72h-1	72h-2	72h-3
GjTrihelix-1	4.4	2.68	2.12	1.6075	1.39	1.315	1.01	0.89	0.7925	2.0625	1.32	1.0725	2.31	2.13	1.995
GjTrihelix-2	3.92	1.58	1.03	2.555	2.36	1.7525	0.6975	0.69	0.675	3.1275	0.63	0.54	1.415	1.13	0.9875
GjTrihelix-3	23.69	21.42	20.07	23.7	22.23	20.01	13.13	11.89	11.39	23.56	21.14	18.25	16.93	16.34	16.32
GjTrihelix-4	0	0.04	0	0.13	0.04	0.01	0.7025	0.14	0.035	0.1425	0.09	0.0525	0.06	0	0
GjTrihelix-5	52.56	50.72	46.19	73.535	65.72	47.6075	60.45	46.08	41.355	80.2725	78.84	59.295	168.92	114.95	95.42
GjTrihelix-6	17.43	10.73	9.46	15.1325	14.96	9.9425	12.955	11.29	9.1	15.6825	13.74	13.1175	13.495	11.95	10.84
GjTrihelix-7	82.99	70.44	43.98	85.0325	83.6	62.3825	70.7725	51.55	45.805	90.93	80.07	73.44	202.825	166.03	120.1
GjTrihelix-8	26.99	22.22	15.6	41.0725	38.14	33.475	36.2575	30.4	21.34	48.7025	48.41	45.77	38.715	38.52	34.5975
GjTrihelix-9	4.45	4.12	3.76	6.165	4.68	4.2525	8.955	7.14	5.6925	6.9175	6.61	4.84	8.82	6.78	6.5325
GjTrihelix-10	23.67	17.35	16.32	19.57	17.59	16.1575	33.5625	27.6	19.965	15.9725	15.92	14.8775	26.61	18.81	15.705
GjTrihelix-11	61.16	39.14	38.86	64.7475	54.81	42.705	17.06	13.31	11.15	59.905	57.7	48.685	27.11	17.9	17.45

Table S6. Detailed information of the 11 *GjTrihelix* genes identified in *Gardenia jasminoides*.

Gene ID	Gene name	Chromosome	Genomic Location (Start-End)	Strand	ORF Length (bp)	Protein Length (aa)	Trihelix Domain Coordinates (aa)	Subfamily
IMPTGJA1N27720_1	*GjTrihelix-1*	chr9	27909070-27911074	+	1740	579	416-503	
IMPTGJA1N28316_1	*GjTrihelix-2*	chr9	5808542-5809782	+	867	288	38-121	
IMPTGJA1N31027_1	*GjTrihelix-3*	chr9	70487016-70490444	+	1167	388	57-150	
IMPTGJA1N33021_1	*GjTrihelix-4*	chr9	9720656-9723592	-	1758	585	128-214; 427-507	
IMPTGJA1N55042_1	*GjTrihelix-5*	chr10	23875995-23876845	-	372	123	27-112	
IMPTGJA1N59694_1	*GjTrihelix-6*	Chr2	9719624-9721944	+	1821	606	125-197; 491-582	
IMPTGJA1N53599_1	*GjTrihelix-7*	chr10	23874819-2387	-	1080	359	186-273	

			5898					
IMPTGJA1N47726_1	*GjTrihelix-8*	Chr8	1703679-1706417	-	2004	667	75-161;483-570	
IMPTGJA1N24434_1	*GjTrihelix-9*	Chr9	58525141-58531730	-	1110	369	102-186	
IMPTGJA1N15413_1	*GjTrihelix-10*	Chr11	43434622-43438683	-	1206	401	85-169	
IMPTGJA1N33773_1	*GjTrihelix-11*	Chr9	71471842-71487932	+	2685	894	800-884	

Table S7. The domain organization diagrams of GjTrihelix.

Gene name	The domain boundary coordinates
GjTrihelix-1	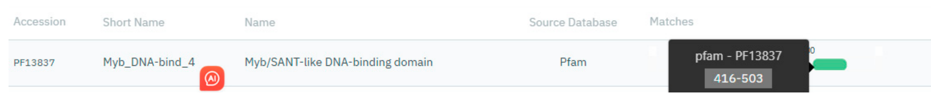
GjTrihelix-2	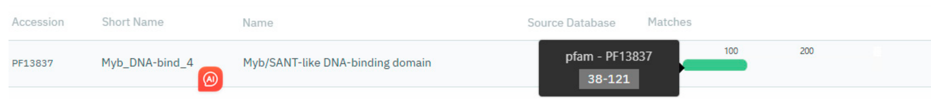
GjTrihelix-3	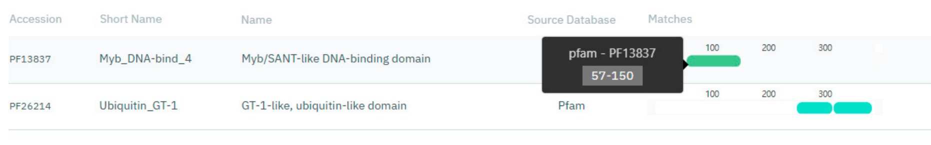
GjTrihelix-4	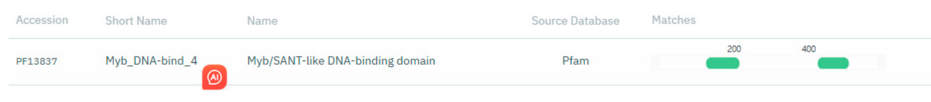
GjTrihelix-5	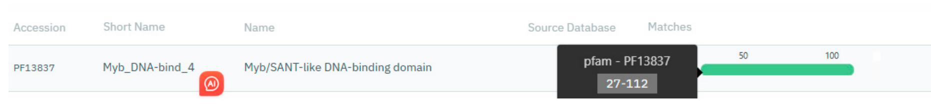
GjTrihelix-6	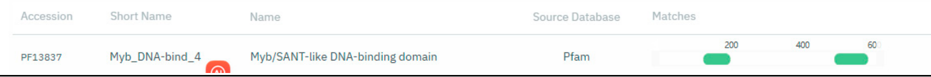
GjTrihelix-7	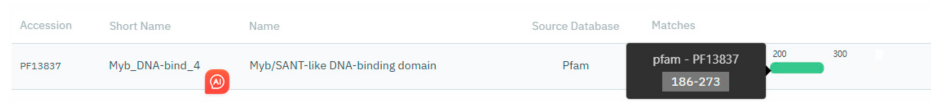
GjTrihelix-8	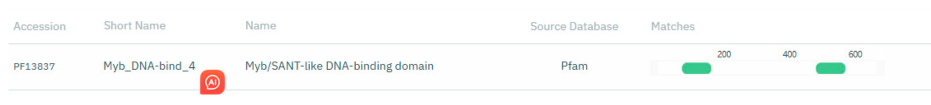
GjTrihelix-9	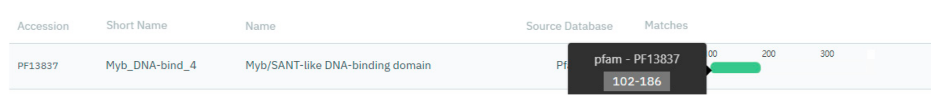
GjTrihelix-10	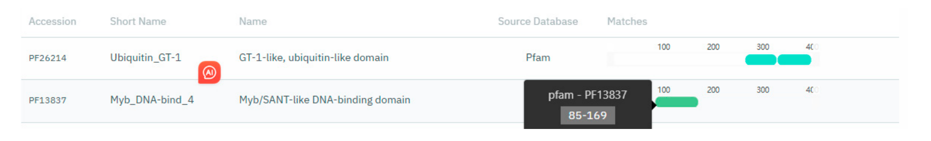

GjTrihelix-1 1	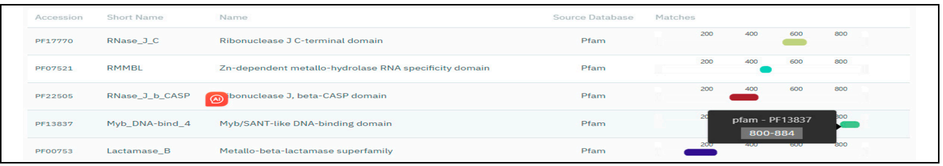

Accession	Short Name	Name	Source Database	Matches
PF17770	RNase_J_C	Ribonuclease J C-terminal domain	Pfam	
PF07521	RMMBL	Zn-dependent metallo-hydrolase RNA specificity domain	Pfam	
PF22505	RNase_J_b_CASP	[illegible]ibonuclease J, beta-CASP domain	Pfam	
PF13837	Myb_DNA-bind_4	Myb/SANT-like DNA-binding domain	Pfam	
PF00753	Lactamase_B	Metallo-beta-lactamase superfamily	Pfam	

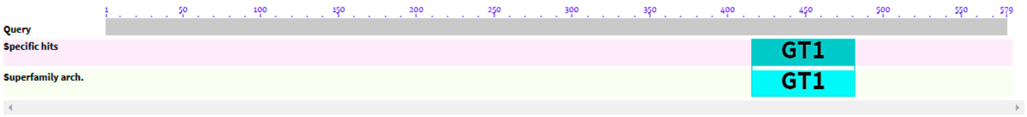

Name	Accession	Description	Interval	E-value
GT1	cd12203	GT1, myb-like, SANT family	416-481	6.02×10^{-27}

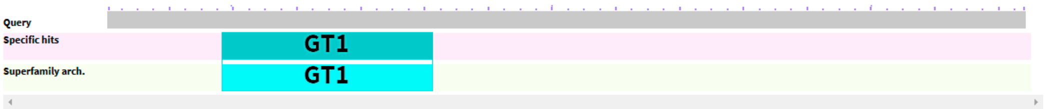

Name	Accession	Description	Interval	E-value
GT1	cd12203	GT1, myb-like, SANT family	37-102	2.04×10^{-24}

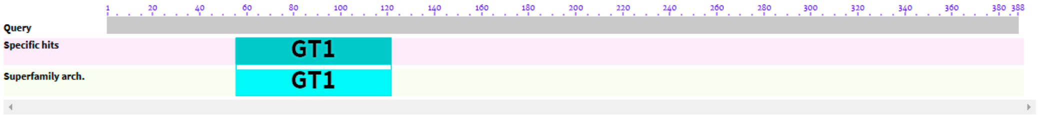

Name	Accession	Description	Interval	E-value
GT1	cd12203	GT1, myb-like, SANT family	56-121	3.78×10^{-27}

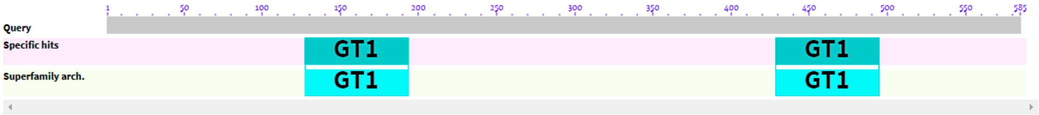

Name	Accession	Description	Interval	E-value
GT1	cd12203	GT1, myb-like, SANT family	429-494	1.46×10^{-20}
GT1	cd12203	GT1, myb-like, SANT family	128-193	2.09×10^{-20}
